# In Vivo Activity Profiling of Biosynthetic Darobactin D22 against Critical Gram-Negative Pathogens

**DOI:** 10.1021/acsinfecdis.4c00687

**Published:** 2024-11-20

**Authors:** Andreas M. Kany, Franziska Fries, Carsten E. Seyfert, Christoph Porten, Selina Deckarm, María Chacón Ortiz, Nelly Dubarry, Swapna Vaddi, Miriam Große, Steffen Bernecker, Birthe Sandargo, Alison V. Müller, Eric Bacqué, Marc Stadler, Jennifer Herrmann, Rolf Müller

**Affiliations:** † Helmholtz Institute for Pharmaceutical Research Saarland (HIPS)−Helmholtz Centre for Infection Research (HZI), Saarbrücken 66123, Germany; ‡ Evotec, Lyon 69007, France; § Evotec, Alderly Park SK10 4TG, United Kingdom; ∥ Helmholtz Centre for Infection Research (HZI), Department Microbial Drugs, Braunschweig 38124, Germany; ⊥ Institute of Microbiology, Technische Universität Braunschweig, Braunschweig 38106, Germany; # Evotec, Marcy L’Etoile 69280, France; ∇ Department of Pharmacy, Saarland University, Saarbrücken 66123, Germany; ○ Deutsches Zentrum für Infektionsforschung (DZIF) e.V., Braunschweig 38124, Germany; ◆ Helmholtz International Lab for Anti-infectives, Saarbrücken 66123, Germany

**Keywords:** darobactins, natural product antibiotic, in vivo infection models, pharmacokinetics, UTI, peritonitis

## Abstract

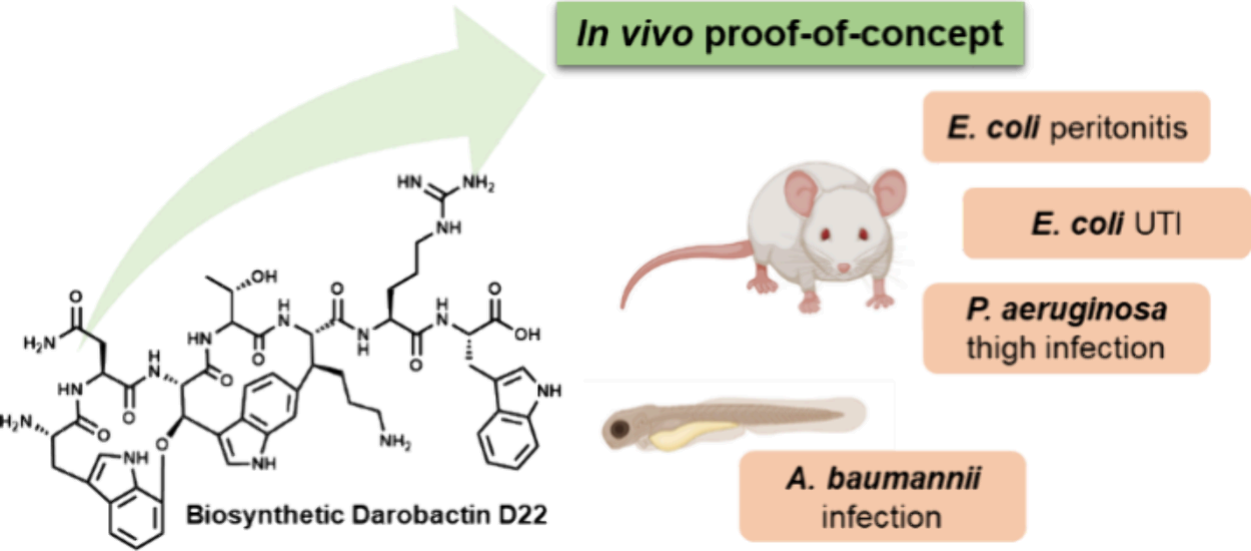

In recent years, naturally occurring darobactins have emerged as a promising compound class to combat infections caused by critical Gram-negative pathogens. In this study, we describe the in vivo evaluation of derivative D22, a non-natural biosynthetic darobactin analogue with significantly improved antibacterial activity. We found D22 to be active in vivo against key critical Gram-negative human pathogens, as demonstrated in murine models of *Pseudomonas aeruginosa* thigh infection, *Escherichia coli* peritonitis/sepsis, and urinary tract infection (UTI). Furthermore, we observed the restored survival of *Acinetobacter baumannii*-infected embryos in a zebrafish infection model. These in vivo proof-of-concept (PoC) in diverse models of infection against highly relevant pathogens, including drug-resistant isolates, highlight the versatility of darobactins in the treatment of bacterial infections and show superiority of D22 over the natural darobactin A. Together with a favorable safety profile, these findings pave the way for further optimization of the darobactin scaffold toward the development of a novel antibiotic.

In the fight against increasing rates of antimicrobial resistance (AMR), there is a particular need for novel antibiotics targeting Gram-negative bacteria.^1−3^ The World Health Organization (WHO) recently presented an updated priority pathogen list categorizing Gram-negative carbapenem-resistant *Acinetobacter baumannii* (CRAB) and Enterobacterales as critical priority pathogens and *Pseudomonas aeruginosa* or *Neisseria gonorrheae* as high priority.^1^ Nonetheless, the pipeline for new antibiotics targeting these pathogens is scarce.^1,3,4^ While the introduction of β-lactam-derived cefiderocol^5^ or sulbactam/durlobactam^6^ represents significant advancements in the field, there is an urge to develop future treatment options based on new chemical entities addressing novel antibacterial targets.^4,7^

A promising new chemical scaffold is darobactins, a novel class of antibiotic natural compounds originally discovered from the entomopathogenic bacterium *Photorhabdus khanii* HGB1456.^8^ Darobactins are ribosomally synthesized and posttranslationally modified peptides (RiPPs) characterized by broad-spectrum activity against Gram-negative pathogenic bacteria. They act on a unique novel target, the transmembrane protein BamA.^8−12^ Inhibition of this outer membrane protein by darobactin results in insufficient folding and insertion of proteins into the outer membrane, eventually leading to cell death.^8,9,12^ As an essential membrane protein that is conserved across bacterial species but absent in humans, BamA is considered a favorable and exciting new antibacterial target.^12−15^ In addition, darobactins bind BamA in the periplasm^9,10,16^ (Figure 1A), thereby circumventing the need to cross both membranes of the Gram-negative cell, which poses a considerable and well-known challenge in targeting these bacteria.^2,14,17^

**Figure 1:**
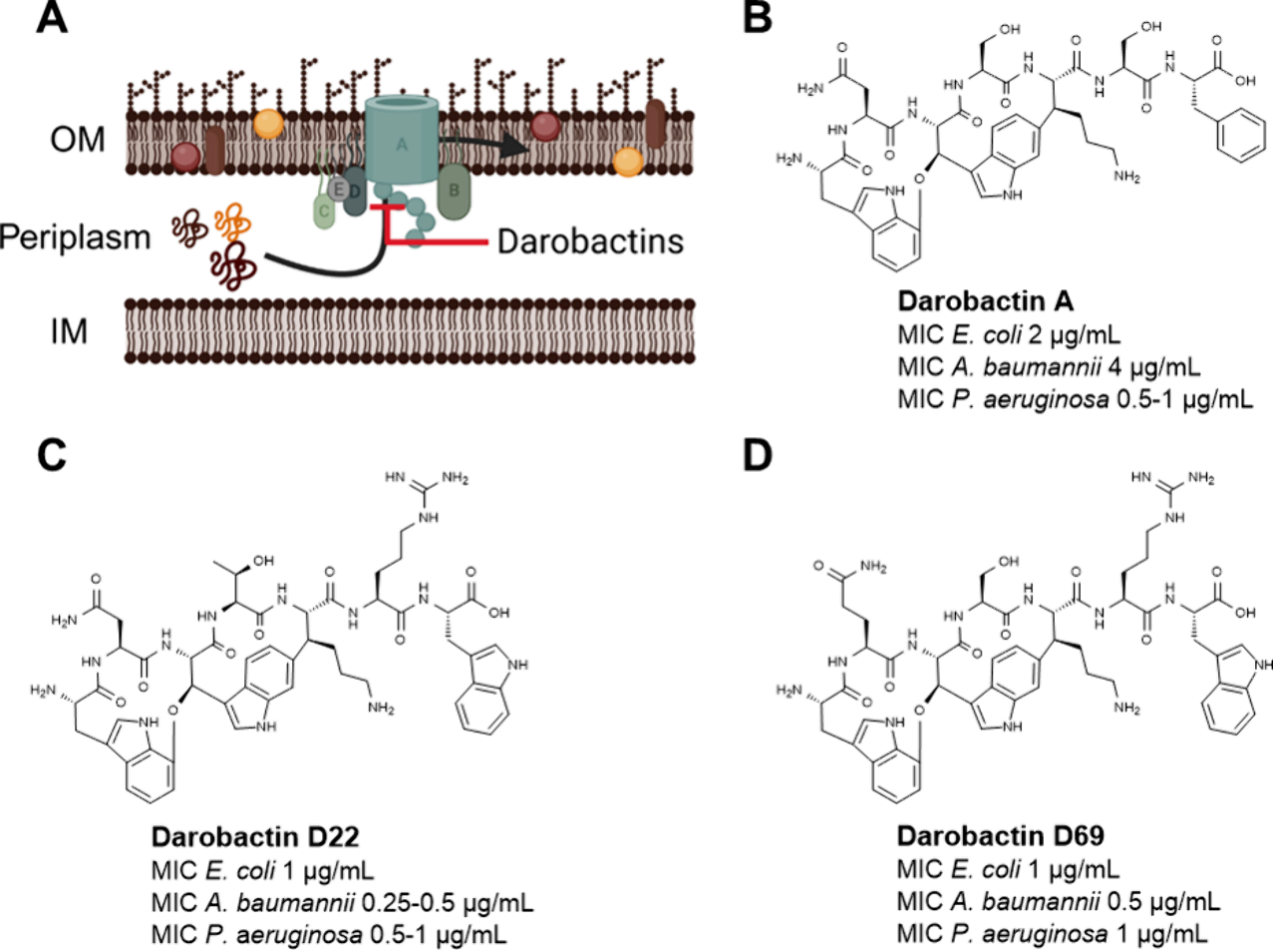
Darobactins target the transmembrane protein BamA in the periplasm by binding to the lateral gate. As a consequence, outer membrane protein folding and integration are impaired, leading to antibiotic activity (A). Structures and activities of the natural compound darobactin A^8^ (B), improved biosynthetic derivatives D22^16^ (C) and D69 (D).^19^ MIC values were determined against *Escherichia coli* ATCC25922, *Acinetobacter baumannii* DSM30008, and *Pseudomonas aeruginosa* PAO1.^16,19^ OM: outer membrane; IM: inner membrane.

In order to expand the structural space and to advance darobactins in the drug discovery pipeline, we have established a heterologous production process enabling access to new derivatives of the natural darobactin A (Figure 1B).^16,18,19^ This approach yielded the biosynthetic frontrunners darobactin 22 (D22) and darobactin 69 (D69) (Figure 1C,D), with improved activity against *A. baumannii*.^16,18,19^ This was accompanied by improved binding to the target as confirmed via microscale thermophoresis and cryogenic electron microscopy, allowing for structure-based optimization.^16,18,19^ Furthermore, darobactin analogues were found to be active against drug-resistant clinical isolates of *A. baumannii* and *P. aeruginosa*.^16,20^ Recently, we determined the in vitro ADMET properties of selected darobactins in preparation for in vivo pharmacokinetic (PK) and pharmacodynamic (PD) studies, observing high metabolic stability and low plasma protein binding.^19^ In the present work, we took the characterization of frontrunner D22 one step further, performing a comprehensive characterization of its in vivo PK and PD. This work aimed at exploring potential target pathogens and indications in vivo. Toward this end, we first measured MIC_90_ against relevant pathogens, particularly in view of the improved activity against *A. baumannii*. Subsequently, we demonstrated activity in a zebrafish embryo infection model with *A. baumannii* and in murine models of *P. aeruginosa* thigh infection, *E. coli* peritonitis/sepsis, and urinary tract infection (UTI). These results unravel, for the first time, the potential of darobactins in the treatment of UTIs, which are caused predominantly by *E. coli* and some other Gram-negative pathogens.^21,22^

## Results

### Production of Darobactins

The production and purification were performed as described in previous studies and according to an adapted protocol (see Experimental Section).^16,19^ In order to obtain the required quantities, particularly of D22 for subsequent in vivo studies, the biosynthetic production was scaled up to 150 L fermenters. The purity of D22 was determined via UV and UHPLC-HRMS analysis, and the compound was subsequently filter sterilized.

### MIC_90_ Determination

Prior to the in vivo profiling, we determined the MIC_50_ and MIC_90_ of D22 in comparison to darobactin A against a selection of Gram-negative pathogens covering *E. coli*, *P. aeruginosa*, *A. baumannii*, and *K. pneumoniae* (Tables 1, S1–S4 and Figure S1). In the case of *E. coli* and *K. pneumoniae*, D22 maintained the activity of darobactin A with MIC_90_ of 2–4 and 4–8 μg/mL, respectively. For *P. aeruginosa*, we saw a moderate, 2–4-fold increase in antibacterial activity, while the MIC_90_ against *A. baumannii* was significantly improved from 64 to 8 μg/mL. Unlike for the other pathogens, the MIC distribution for *A. baumannii* was found to be clearly bimodal (Figure S1D), with the most sensitive subpopulation in the range of <1 μg/mL. This finding is in accordance with the very good activity of D22 against clinical isolates observed before.^16^

**Table 1:** MIC_50_ and MIC_90_ of D22 against Clinical Isolates of *Escherichia coli*, *Klebsiella pneumoniae*, *Pseudomonas aeruginosa*, and *Acinetobacter baumannii* (*n* = 21–31) Compared to Darobactin A^a^

MIC_90_ (μg/mL)	darobactin A		D22	
	MIC_50_ (μg/mL)	MIC_90_ (μg/mL)	MIC_50_ (μg/mL)	MIC_90_ (μg/mL)
*E. coli*	1	2	1–2	2–4
*P. aeruginosa*	16	32	8	8–16
*A. baumannii*	16	64	2	8
*K. pneumoniae*	2	4	2–4	4–8

a Individual MIC values are given in Tables S1–S4. MIC_50_ and MIC_90_ determinations were performed in technical duplicates.

### Zebrafish Embryo Model of *A. baumannii* Infection

Since D22 showed a significant improvement in activity against multidrug-resistant *A. baumannii*, including potent killing of CRAB (MIC ≤ 0.5 μg/mL^16^), we sought to confirm the superiority over darobactin A in an in vivo model of infection. In an effort to collect initial in vivo activity data, we employed an experimental model of *A. baumannii* infection using zebrafish embryos, considering the challenges of establishing murine infection models with this pathogen.^23^ This allowed us to compare different derivatives in vivo and to demonstrate a proof of concept (PoC) for the optimized darobactins with respect to the treatment of *A. baumannii* infection.

Inspired by previous work,^24^ we refined the zebrafish embryo model with regard to the assessment of in vivo drug efficacy. To this end, we established an *A. baumannii* infection model in zebrafish embryos at various developmental stages by microinjecting rising doses of GFP-tagged *A. baumannii* into the caudal vein or yolk sac (Figure S2). Upon monitoring the survival of infected embryos, we observed dose- and site-dependent mortality. Furthermore, the developmental stage at the time of infection seems to have a major impact on zebrafish resistance to *A. baumannii* infection. Embryos showed hypersusceptibility to the bacterial infection when they were infected at 1 day post-fertilization (dpf) as compared to 2 dpf, irrespective of the infection site. At 1 dpf, as few as 50 colony-forming units (CFUs) were sufficient to cause a lethal infection with mortality rates reaching 90 and 70% for yolk sac infection and caudal vein infection, respectively. By contrast, at 2 dpf, higher infectious inoculums were required to reach comparable mortality rates; e.g., for yolk sac infection, a minimal dose of 2500 CFU was needed to reach the same mortality as 50 CFU at 1 dpf. Consistent with yolk sac infection, high doses of 2500 to 5000 CFU were required to achieve a lethal caudal vein infection. Neutrophils were previously reported to be the dominant phagocyte responders in the defense against *A. baumannii* infection in zebrafish embryos.^24^ However, functional neutrophils only become present at 30–48 h post-fertilization (hpf)^25,26^; thus, at 1 dpf, the first line of defense against the pathogen is missing, rendering the embryos hypersusceptible to a lethal disease. Taking these findings into consideration, we settled for caudal vein infection with 2,500 CFU at 2 dpf for further treatment studies, as microinjection into the caudal vein leads to a systemic infection, resembling bacteremia in humans.

As we have previously shown that the route of drug administration has a great impact on the in vivo activity of drugs,^27^ we decided to administer darobactins via microinjection into the caudal vein to ensure systemic distribution inside the embryonic body and to avoid false negatives as a consequence of insufficient uptake following waterborne exposure. In order to determine an appropriate dose for future comparison of different derivatives, we performed a dose titration of D22 in embryos systemically infected with 2500 CFU of *A. baumannii* and found the minimal effective dose to be 10 mg/kg (Figure S3). Thus, the treatment dose for all tested antibiotics was set to 10 mg/kg for a head-to-head comparison.

**Figure 2:**
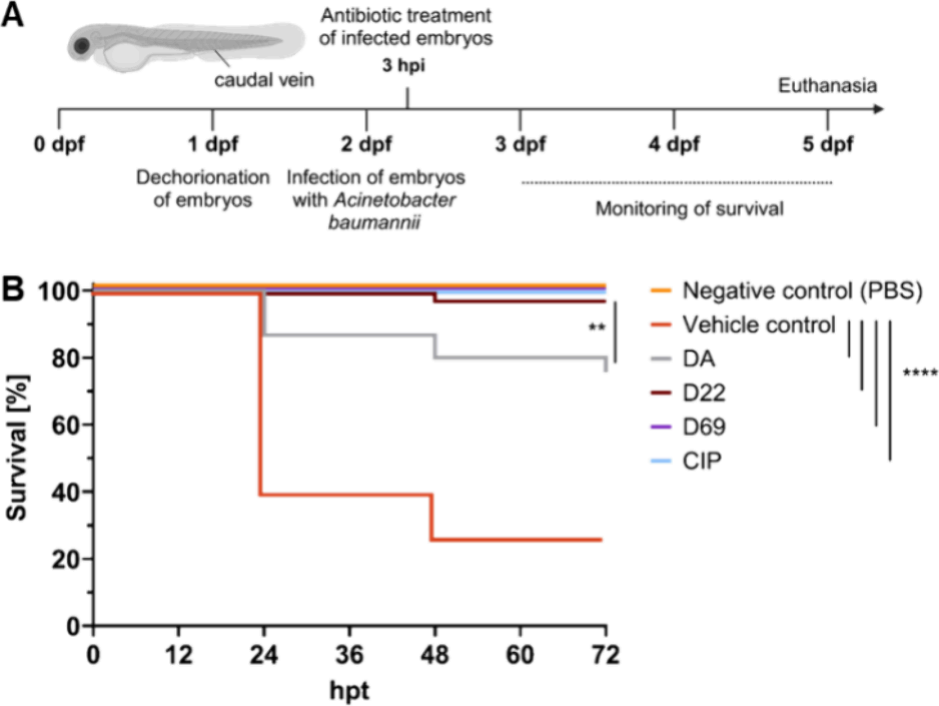
Darobactin derivatives are highly active in a zebrafish embryo model of *Acinetobacter baumannii* infection. Experimental design of the *A. baumannii* infection model with infection and treatment time points (A). Survival curves of zebrafish embryos that were systemically infected with *A. baumannii* ATCC17978 and treated 3 hpi with 10 mg/kg of darobactin derivatives via microinjection into the caudal vein. CIP (10 mg/kg) served as a comparative treatment. Infected, PBS-treated embryos served as positive (vehicle) control, whereas noninfected PBS-injected embryos served as negative control (B). Survival curves represent 3 independent experiments with 15 embryos per group each. Comparison between survival curves were made using the log rank (Mantel-Cox) test (*p* < 0.01: **, *p* < 0.0001: ****). Dpf: days post fertilization; hpi: hours post infection; hpt: hours post treatment; CIP: ciprofloxacin.

All tested darobactin derivatives exhibited significant in vivo activity (*p* < 0.0001) against *A. baumannii* as reflected by higher survival rates compared to the vehicle control (Figure 2). While the natural darobactin A increased survival up to >75%, the biosynthetic frontrunners D22 and D69 completely cleared *A. baumannii* infection within the zebrafish embryos, confirming the higher potency observed in vitro (Table S5) and also in vivo (*p* < 0.01). Noteworthy, D22 and D69 showed efficacies in clearing the infection equivalent to that of the clinically used ciprofloxacin taken as the reference antibiotic in this zebrafish infection model. These promising findings provided a good starting point for the characterization of D22 in murine models of infection.

### In Vivo Pharmacokinetic Studies in Mice

**Figure 3:**
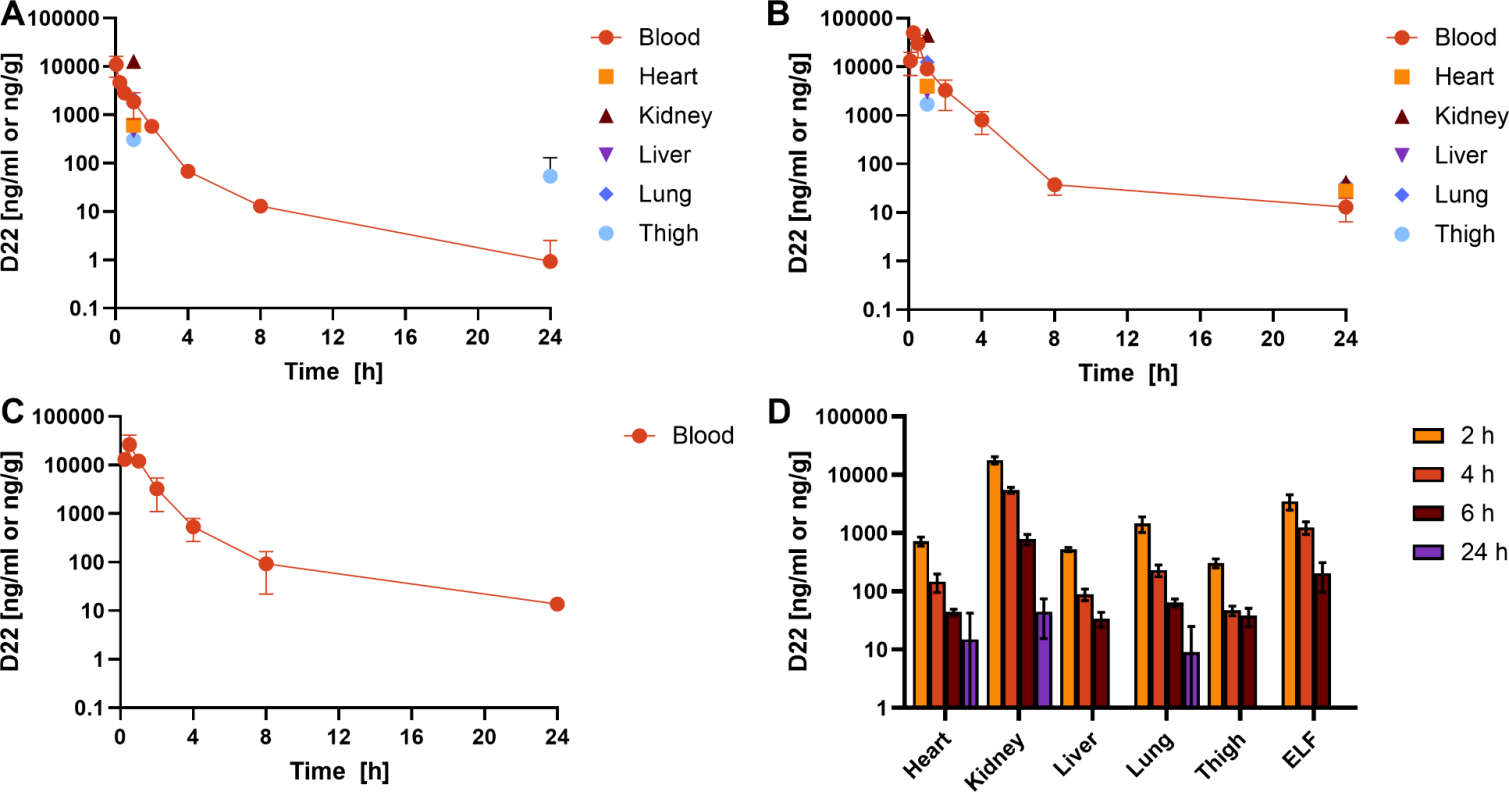
Pharmacokinetic studies in C57BL/6 mice. Blood and tissue levels (1, 24 h) of D22 after single intravenous (5 mg/kg, A) and intraperitoneal administration (20 mg/kg, B). Blood levels of D22 were after single subcutaneous administration (20 mg/kg, C). Levels of D22 in the heart, kidney, liver, lung, thigh tissue, and ELF after subcutaneous administration (20 mg/kg, D). Corresponding tissue/fluid levels are given in Table S5. Concentrations are given in ng/mL for blood and ELF or in ng/g for heart, kidney, liver, lung, and thigh tissue, representing means ± SD for 3 animals each. ELF: epithelial lining fluid.

In preparation for murine infection models, we subjected D22 to detailed PK studies in mice. 5 mg/kg was applied via intravenous (IV) administration (Figure 3A) and 20 mg/kg via subcutaneous (SC) and intraperitoneal (IP) administration (Figure 3B–D). Additional groups received 5 mg/kg intratracheally (IT) and 20 mg/kg orally (PO, Figure S4). The resulting PK parameters are given in Tables 2 and S6–S8. Generally, half-lives were low (≤1 h), as observed previously for darobactin A.^8^ Following administration of an IV bolus, a biphasic blood profile (half-life of 0.6–3.5 h) was observed with a predominant half-life of 0.6 h. The longest half-life was found after SC administration (1 h). For this route of administration, a detailed analysis of tissue levels over time revealed fast distribution into the kidney with tissue concentrations up to 17.9 μg/g (Figure 3D). Similar observations were made when D22 was dosed IV and IP (12.9 and 45.1 μg/g of kidney tissue after 1 h, respectively). High levels of unmodified compound were detected in urine (Table S7), which is in agreement with the high polarity of the compound, the high metabolic stability, and low plasma protein binding observed before.^19^ As anticipated, clearance was predominantly renal (55% of total clearance, Table S7) and less than 1% of the administered dose was recovered in feces (Table S8). Additionally, significant compound levels were detected in the thigh muscle.

**Table 2:** Blood Pharmacokinetic Parameters of D22 after Different Routes of Administration^a^

route (mg/kg)	*C*_0_ (ng/mL)	*C*_max_ (ng/mL)	*t*_1/2_ (h)	*t*_max_ (h)	AUC_0-last_ (h^b^ ng/mL)	*t*_last_ (h)	AUC_0–inf_ (h* ng/mL)	*F* (%)
IV 5	13,740		0.6^α^/3.5^β^		5946		5947^α^/5951^β^	NA
SC 20		26,567	1.0	0.50	29,784	24	29,805	>100^b^
IP 20		50,533	0.8	0.25	38,157	24	38,173	>100^b^
IT 5		872	ND	0.08	2243	24	ND	37.7
PO 20		33.2	ND	0.50	147	24	ND	0.62

a For IV administration, *t*_1/2_ and AUC_0–inf_ are given for α- and β-phases of the biphasic elimination. C_0_: concentration at t = 0, *C*_max_: maximum concentration; *t*_1/2_: half-life, *t*_max_: time at which *C*_max_ is reached; AUC: area under the curve, t_last_: time of the last sample; F: bioavailability (relative to IV group).

b Values could not be determined unambiguously due to nonlinear dose dependency using different routes of administration.

As expected based on the physicochemical properties of the compound, oral bioavailability was negligible (Table 2 and Figure S4A). In order to achieve oral bioavailability, modification of the darobactin structure, rendering it less polar, may be envisioned. This should further have an impact on plasma half-life by increasing the low PPB, which was also postulated by Böhringer et al.^28^ since higher PPB is known to reduce clearance.^29^ IT dosing led to the expected high lung and epithelial lining fluid (ELF) levels (Figure S4B), while lung exposure was also observed after systemic dosing.

These findings encouraged us to assess the in vivo efficacy of D22 after systemic administration. Based on the favorable exposure in the relevant compartments, we chose thigh and urinary tract infections in addition to bloodstream infection.

### *P. aeruginosa* Thigh Infection Model

**Figure 4:**
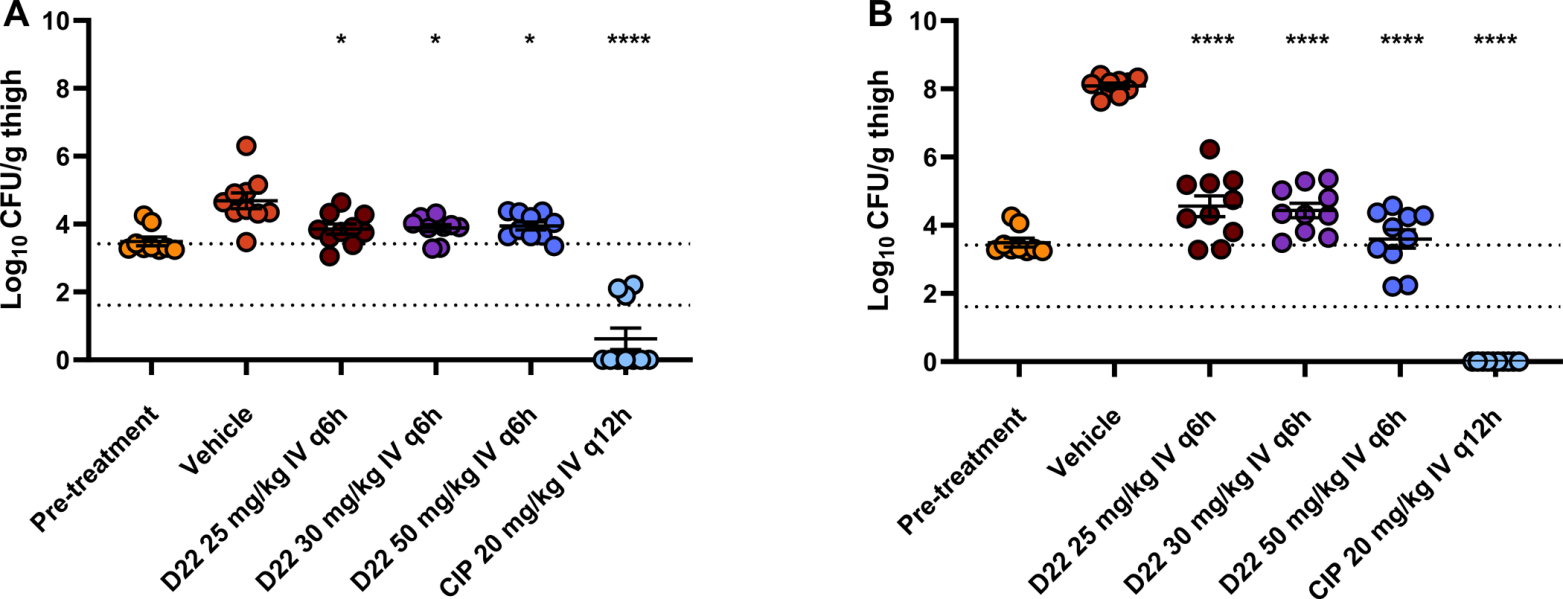
Murine neutropenic thigh infection model using *Pseudomonas aeruginosa* PAO1. Bacterial burden in thigh muscle of CD-1 mice at 8 hpi (A) and 25 hpi (B). Dashed lines indicate the detection limit (1.4 log of CFU/g) and stasis level (3.4 log of CFU/g). Eight animals were used for the pretreatment group and 10 animals for vehicle and treatment groups. Mean ± SEM is depicted and significant differences vs. vehicle are indicated: ****: *p* < 0.0001, *: *p* < 0.05 (ANOVA with Dunnett’s multiple comparison test). CFU: colony-forming unit; CIP: ciprofloxacin; hpi: hours post infection.

Having demonstrated in vivo PoC against *A. baumannii* in the zebrafish embryo infection model described earlier, we sought to expand the spectrum of our in vivo studies to the priority pathogen *P. aeruginosa*. Encouraged by the good exposure of D22 in the thigh as seen in the PK study, we ran a neutropenic thigh infection model, which is widely used in anti-infective drug discovery and development.^30−32^ Mice were rendered neutropenic using cyclophosphamide on days −4 and −1 followed by intramuscular infection with *P. aeruginosa* PAO1. Bacterial load in the thigh was determined 8 and 25 h after infection (Figure S5). D22 was administered at 25, 30, and 50 mg/kg IV q6h. At 8 h, after the mice had received the second dose of D22, a moderate, but significant reduction of bacterial burden in the muscle compared to the vehicle control was observed (Figure 4A) with no difference between the applied doses (Δlog CFU/g –0.74 to –0.83), while the control antibiotic ciprofloxacin (20 mg/kg) suppressed bacterial burden below stasis (Δlog CFU/g –4.06). The effect of D22 was more pronounced after 25 h (Figure 4B) with the highest dose of 50 mg/kg reducing the bacterial burden approximately to the stasis level (Δlog CFU/g –4.50). Contrary to the 25 and 30 mg/kg dosing groups, there was no significant bacterial growth observed between 8 and 25 h in both 50 mg/kg dosing group and the control group. Administration of single doses up to 75 mg/kg IP only marginally reduced bacterial burden compared to vehicle control at 25 h (Figure S6). Imai et al. also observed an improved reduction of *E. coli* levels in the thigh after repeated dosing of darobactin A.^8^ These findings suggest that more frequent dosing would be necessary to reach efficacy in this model, in line with the determined in vivo *t*_1/2_ of ≤1 h. Strategies to increase plasma half-life as outlined above may further be beneficial for tissue distribution and contribute to reducing the bacterial load below stasis. Yet, we consider this finding an encouraging PoC for the treatment of *P. aeruginosa* infections and a valuable starting point for the optimization of efficacy in relevant models taking into account that *P. aeruginosa* is responsible for hospital-acquired and ventilator-associated pneumonia (HAP/VAP)^33^ as well as bloodstream and urinary tract infections.^22,34^

As a next step, we explored the potential of D22 in the latter two indications. In view of the more favorable in vitro potency and availability of suitable animal models, we characterized the in vivo efficacy against *E. coli*.

### *E. coli* Peritonitis Model

**Figure 5:**
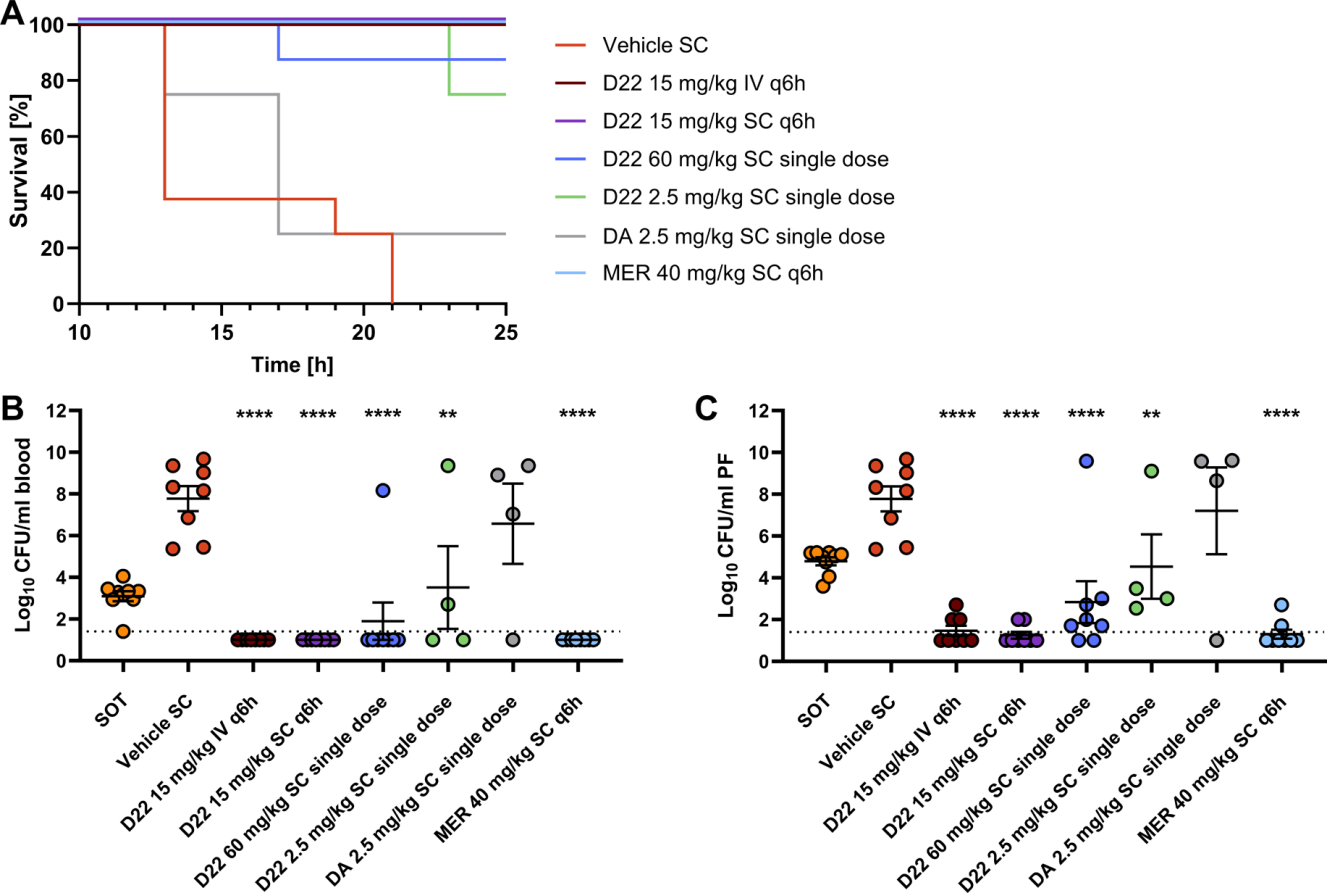
Murine peritonitis model with *Escherichia coli* 106–09. Survival of mice after treatment with D22, MER, or vehicle. A clinical score of 3 was used as a surrogate marker for death (A). Colony counts in blood (B) and PF, (C) after completed treatment with D22, MER, or vehicle. Samples with no detectable bacteria are depicted at 1.0 Log CFU. The detection limit is indicated at 1.4 Log CFU. Samples for CFU determination were taken terminally or at the humane end point (*t* = 17–25 h for D22 60 mg/kg; 23–25 h for D22 2.5 mg/kg and 13–25 h for DA 2.5 mg/kg). Eight animals were used per group except for the 2.5 mg/kg dosing groups with 4 animals each. Mean ± SEM is depicted and significant differences vs. vehicle are indicated: ****: *p* < 0.0001, **: *p* < 0.01 (ANOVA with Dunnett’s multiple comparison test). Δlog CFU/mL is given in Table S10. MER: Meropenem; PF: peritoneal fluid; SOT: start of treatment; CFU: colony-forming unit.

In order to establish an in vivo PoC in a more severe infection model, we evaluated the efficacy of D22 in a mouse model of *E. coli* peritonitis. D22 was shown to be efficacious against several clinical isolates, including gentamicin-resistant ones (Table S9), and we subsequently aimed to extend this result to in vivo activity against extended-spectrum β-lactamases-producing (ESBL) *E. coli* 106-09 (MIC 0.5–1 μg/mL).^35^ Mice were infected IP (Figure S7). When applied in 4 doses of 15 mg/kg each, D22 increased survival to 100% both after IV and SC administration (Figure 5A). The infection was fully cleared in blood, and bacterial burden significantly reduced below the stasis level in the peritoneal fluid (PF, Figure 5B,C and Table S10). The same total daily dose applied as a single SC dose increased survival to 87% and reduced bacterial burden below the stasis level. Inspired by previous results on darobactin A, we included a lower dose of 2.5 mg/kg of D22 that led to 75% survival, while the same dose of darobactin A only reached 25% and led to increased bacterial growth in both blood and PF compared to the inoculum. This improved survival rate could be due to the use of a different *E. coli* isolate compared to Imai et al.,^8^ but here, we could clearly show superiority of D22 vs darobactin A. CFU reduction in blood and PF was significant for all tested doses of D22. Bacteria were not fully cleared in the single dose groups and levels, but even the lower dose of 2.5 mg/kg reduced the bacterial burden to stasis. This observation hints at a need for repeated dosing, yet the difference between single and repeated dose treatment is not as pronounced as in the thigh infection model shown above, potentially due to the better availability of D22 in blood compared with muscle tissue. The effects observed at 2.5 mg/kg in comparison to 60 mg/kg suggest a minor relevance of *c*_max_/MIC in the in vivo efficacy of D22, serving as a starting point for more detailed dose-fractionation studies in the future to identify the PK/PD driver for D22. Overall, the results provide a PoC for the biosynthetic derivative D22 against severe infections and confirm its superiority over darobactin A in vivo, as seen in the zebrafish model described above.

### *E. coli* Urinary Tract Infection Model

**Figure 6:**
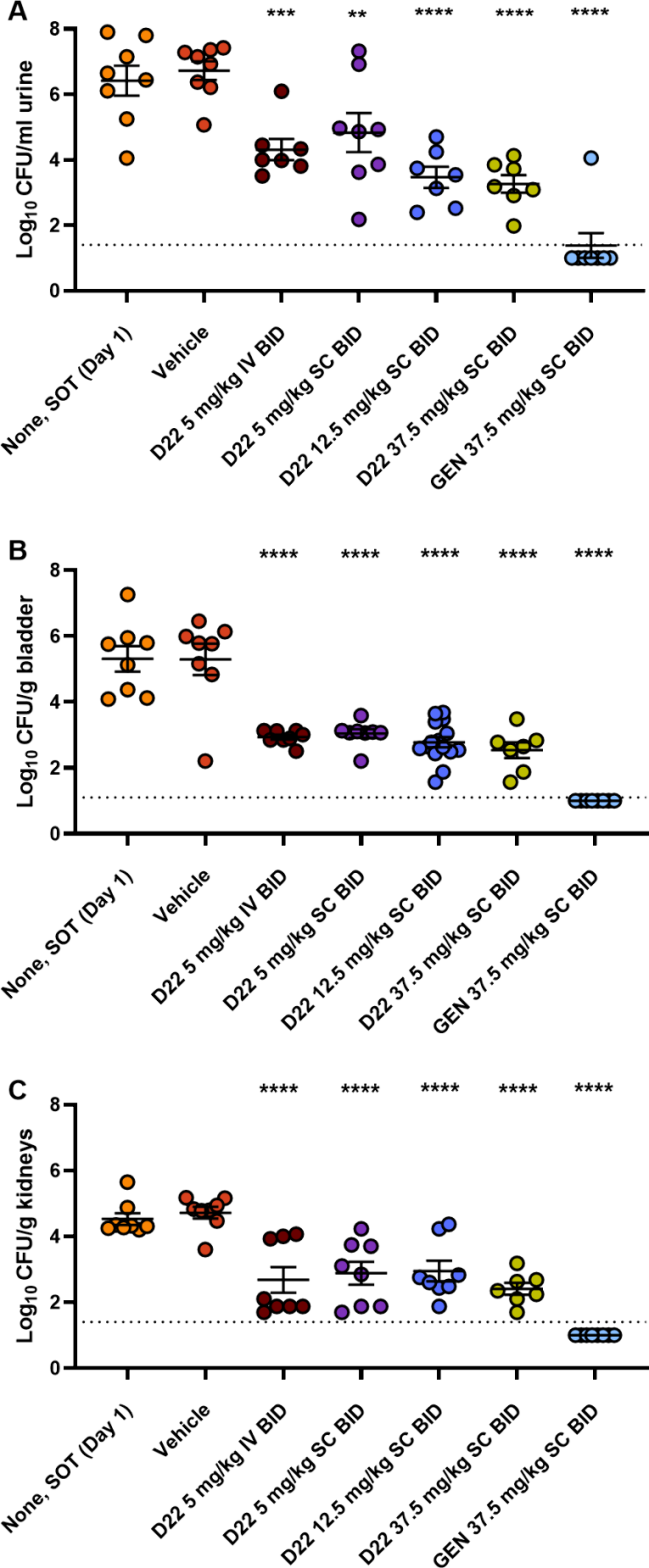
In vivo model of *Escherichia coli* urinary tract infection. C3H/HeNHsd mice were infected with *E. coli* isolate C175-94 via a catheter and treated with D22 and GEN in sterile saline following a BID dosing scheme on days 1, 2, and 3 post inoculation. CFUs were determined on day 1 or day 4 in urine (A), bladder (B), and kidney (C). Samples with no detectable bacteria are depicted as 1.0 Log CFU. Dashed lines indicate the detection limit of 1.4 Log CFU. Eight animals were used per group. Mean ± SEM is depicted and significant differences vs. vehicle are indicated. ****: *p* < 0.0001, ***: *p* < 0.001, **: *p* < 0.01 (ANOVA with Dunnett’s multiple comparison test). GEN: gentamicin; CFU: colony-forming unit; SOT: start of treatment.

The good coverage of *E. coli* (MIC_90_ 2–4 μg/mL, unimodal distribution, Tables 1 and S1 and Figure S1), which is the leading cause of UTI,^22^ together with the observed high urine and kidney levels in the murine PK prompted us to study D22 in an ascending *E. coli* UTI model representative of complicated UTI (cUTI). Mice were infected with the clinical isolate *E. coli* C175-94^36^ (MIC 0.125–0.25 μg/mL, Table S9 and Figure S8). Despite the slightly higher AUC in blood after IP dosing (Table 2), we opted for SC administration as this led to a higher urine concentration of 328 μg/mL at 20 mg/kg (Table S7), corresponding to ∼1300- to 2600-fold vs MIC. In view of the fast excretion of D22 and the lessons learned from the thigh infection model described earlier, a bi-daily dosing scheme was applied with doses ranging from 5 to 37.5 mg/kg. One IV dosing arm at 5 mg/kg was included for comparison. Mice were treated for 3 days post-inoculation, and the bacterial burden was determined on day 4. D22 significantly reduced bacterial burden in urine, bladder, and kidney in all treatment groups (Figure 6). The effect was found to be dose-dependent in urine (Figure 6A) and the dose of 37.5 mg/kg resulted in a significant CFU reduction in urine (Δlog CFU/mL –3.5), bladder (Δlog CFU/g –2.8), and kidney (Δlog CFU/mL –2.3). No major difference was observed between the IV and SC treatments. The positive control gentamicin resulted in a reduction of bacterial burden below stasis level in all tissues, despite the slightly higher MIC. This might be due to the more favorable pharmacokinetic properties of this antibiotic, as aminoglycosides are characterized by high renal tissue concentrations, yet are also known to cause nephrotoxicity.^37,38^ Taken together, these results highlight that darobactins have the potential to combat clinical UTIs.

### In Vitro Safety Assessment

To complement the promising data set on the in vivo PK and PD properties of D22, we expanded the characterization of its safety profile. We demonstrated previously that along with other biosynthetic darobactin derivatives, D22 is nontoxic against HepG2 cells (CC_50_ > 37 μg/mL). This finding was now confirmed for D22 across a broad range of cell lines, including kidney cell line HEK293 (Table S11). In addition to this, we could demonstrate that neither darobactin A nor D22 leads to hemolysis of human blood cells when tested at concentrations up to 1 mg/mL (Figure S9). Furthermore, we tested D22 in the SafetyScreen44 panel. Notably, at a concentration of 10 μM, no off-target was affected at >25% except for monoamine oxidase A (MAO-A) where control binding was inhibited by 67 ± 13% (Figures S10 and S11). Subsequently, we performed follow-up activity assays revealing only moderate inhibition at 100 μM D22 for both MAO-A (39 ± 13%) and MAO-B (36 ± 6%, Figure S12). In light of the potent antibacterial activity of D22, we do not consider this finding critical at this stage. Overall, D22 displays an excellent safety profile in the assays conducted thus far, underlining its potential for future drug development.

## Discussion

The overall aim of this work was to provide in vivo PoC for non-natural darobactins, particularly the biosynthetic derivative D22. With regard to *A. baumannii*, we have demonstrated for the first time that D22 is capable of killing this critical pathogen in vivo. In particular, CRAB has emerged as a major cause of healthcare-associated infections, especially in intensive care units (ICUs). CRAB infections comprise hospital-acquired septicemia, VAP, and UTI.^39^ By applying a zebrafish embryo infection model, we demonstrated the potential of D22 for the treatment of this critical priority pathogen. These findings encourage further in vivo characterization of D22 in murine models of *A. baumannii* infection. In view of the observed bimodal MIC distribution for D22 against *A. baumannii*, these studies should be complemented by an additional in vitro activity assessment, including a larger panel of strains. For future in vivo studies, inhalative administration seems feasible in view of the very good water solubility of D22, or alternatively the use of systemic dosing, e.g., for the treatment of bloodstream infections.

Indeed, there is an increasing prevalence of bloodstream infections caused by Gram-negative bacteria that can lead to severe and potentially fatal sepsis.^40^ With more than 30 million infections globally per year and increasing rates of resistance, sepsis was declared a global health priority by the WHO and requires novel treatment options.^40,41^ Importantly, the most relevant causative pathogens of Gram-negative bacteremia (*E. coli*, *K. pneumoniae*, *P. aeruginosa*, and *A. baumannii*) are within the spectrum of darobactins, highlighting their potential to tackle this critical infection.^34^ In line with this observation, we have first confirmed that D22 shows efficacy in an *E. coli* peritonitis model along with higher survival rates compared to darobactin A.

Capitalizing on the generated in vivo mouse PK data, we further conducted a *P. aeruginosa* thigh infection model and an *E. coli* UTI model. The measured thigh exposure indeed led to a significant reduction of the bacterial burden, providing a PoC for the treatment of *P. aeruginosa* infections in vivo in this translational model. In vivo efficacy was further confirmed for the treatment of UTI, a disease for which there is a high medical need to develop alternative treatments in view of increasing rates of antibiotic resistance.^21,22,42^ A key benefit of D22 in this regard is its favorable activity against *E. coli* (including resistant isolates), the major causative pathogen of UTIs.^21,22^ While uncomplicated UTI is predominantly caused by *E. coli* (>75%),^22^ the treatment of complicated UTI (cUTI) requires broad-spectrum antibacterial activity as additional pathogens are involved in the pathogenesis, in particular in healthcare settings. As for bacteremia, D22 also addresses the most relevant Gram-negative bacteria in cUTI (*E. coli*, *K. pneumoniae*, and *P. aeruginosa*), with the exception of *Proteus* spp.^21,22^ Additionally, in view of the in vivo activity against *A. baumannii* presented herein, we are optimiztic that UTIs caused by *A. baumannii* may also respond well to treatment with darobactins. The PK study further confirmed that D22 is exposed in both urine and kidney which is required for efficient treatment of UTI.^37^ This led to the first in vivo PoC for darobactins in this type of infection described so far, with significant effects in urine, bladder, and kidney tissue after systemic administration. Considering the lack of oral bioavailability, at this stage treatment would be restricted to severe infections in hospitals by IV administration. An optimization goal in this regard is to increase PPB and thereby plasma half-life, potentially allowing for less frequent dosing and improved tissue penetration.

## Conclusions

The development of new compounds able to tackle the antibiotic resistance crisis requires, on top of a comprehensive characterization of the in vitro activity, systematic profiling of the PK and PD properties in vivo. In the present work, we provide such a characterization for D22, a biosynthetic darobactin analogue. In particular, we have confirmed its broad coverage of key Gram-negative pathogens and demonstrated significant exposure in mice by the IV, SC and IP routes. We then established in vivo PoC regarding the efficacy (by IV and SC) of D22 against an *A. baumannii* infection in zebrafish embryos and in a *P. aeruginosa* thigh infection model as well as in *E. coli* UTI and peritonitis models. Together with the excellent in vitro safety profile observed, these findings bode well for the potential of D22 in treating key clinical infections. In summary, we consider the presented results a promising starting point to guide the translation of darobactins into an innovative solution to fight antimicrobial resistance.

## Methods

Experimental procedures are provided in the Supporting Information. All animal models were performed in accordance with all national or local guidelines and regulations. These and the relevant approving committees are specified in the Supporting Information.

## Supporting Information Available

MIC distribution (Figure S1); Zebrafish resistance (Figure S2); dose titration of darobactin D22 (Figure S3); pharmacokinetic studies (Figure S4); experimental layout of the neutropenic thigh infection model (Figure S5); murine neutropenic thigh infection model applying QID dosing (Figure S6); experimental layout of the peritonitis model (Figure S7); experimental layout of the UTI model (Figure S8); hemolysis assay (Figure S9); SafetyScreen44 histogram (Figures S10 and S11); functional inhibition assays (Figure S12); MIC of D22, ciprofloxacin and colistin (Tables S1–S4); MIC of darobactins against *A. baumannii* (Table S5); tissue levels and tissue-to-blood ratios (Table S6); urine concentrations and renal clearance calculation (Table S7); feces concentration and clearance calculation (Table S8); MIC of D22 and gentamicin (Table S9); difference in bacterial load in blood and PF (Table S10); darobactin D22 is nontoxic against a range of human cell lines (Table S11); and materials and methods (PDF)

## Abbreviations Used

(c)UTI: (complicated) urinary tract infection
AMR: antimicrobial resistance
RiPP: ribosomally synthesized and posttranslationally modified peptide
PoC: proof of concept
dpf: days post fertilization
CFU: colony-forming unit
hpf: hours post fertilization
MIC_90_: lowest concentration that inhibits growth of 90% of tested isolates
MIC_50_: lowest concentration that inhibits growth of 50% of tested isolates
CC_50_: concentration at which cell viability is reduced by 50%
MAO: monoamine oxidase
CRAB: carbapenem-resistant *A. baumannii*
ICU: intensive care unit
VAP: ventilator-associated pneumonia
