# Supplementary material for: In Vivo Activity Profiling of Biosynthetic Darobactin D22 against Critical Gram-Negative Pathogens
Source: ACS Infect Dis. 2024 Nov 20;10(12):4337–46. doi: 10.1021/acsinfecdis.4c00687 (PMC11650638; doi:10.1021/acsinfecdis.4c00687)
Supplement: Supplementary file 1 — id4c00687_si_001.pdf [file id4c00687_si_001.pdf]

Supporting Information

In Vivo Activity Profiling of Biosynthetic Darobactin D22 Against Critical Gram-negative Pathogens

Andreas M. Kany, Franziska Fries, Carsten E. Seyfert, Christoph Porten, Selina Deckarm, María Chacón Ortiz, Nelly Dubarry, Swapna Vaddi, Miriam Große, Steffen Bernecker, Birthe Sandargo, Alison V. Müller, Eric Bacqué, Marc Stadler, Jennifer Herrmann, Rolf Müller

Table of Contents

Supporting Figures S3
Supporting Tables.......... S9
Materials and Methods S14
References S21

Supporting Figures

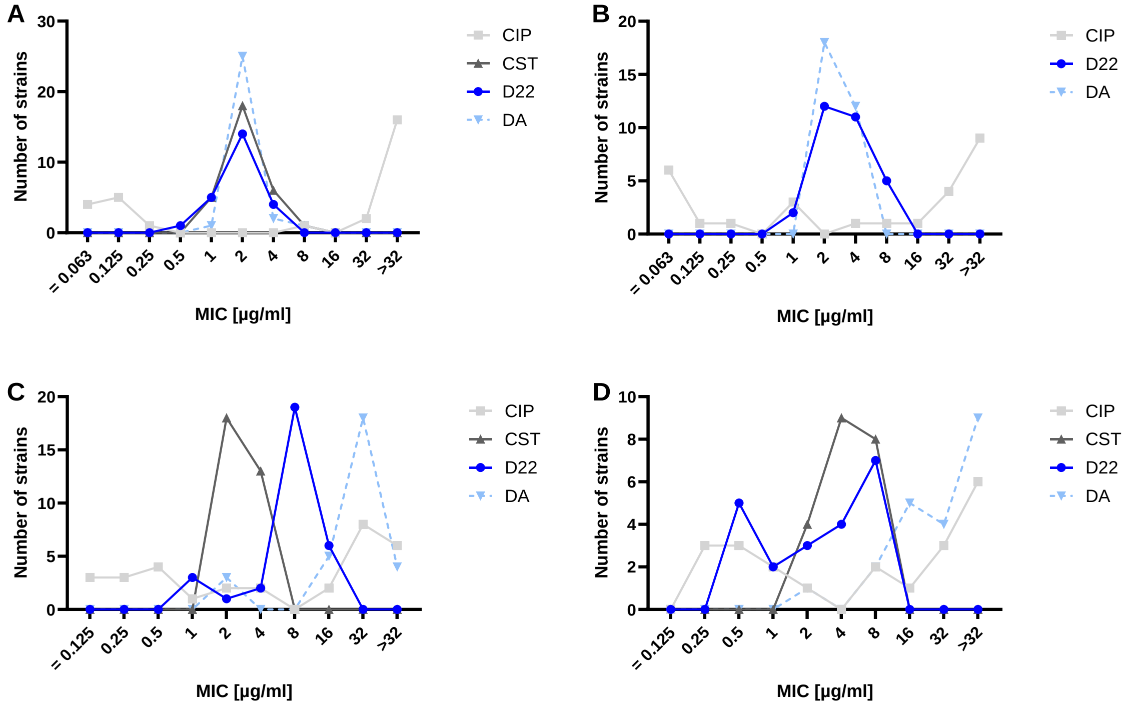

Figure S1. MIC distribution of D22 and DA against *Escherichia coli* (A, n = 30), *Klebsiella pneumoniae* (B, n = 30), *Pseudomonas aeruginosa* (C, n = 31) and *Acinetobacter baumannii* (D, n = 21). MIC determination was performed in technical duplicates. CIP: ciprofloxacin; CST: colistin.

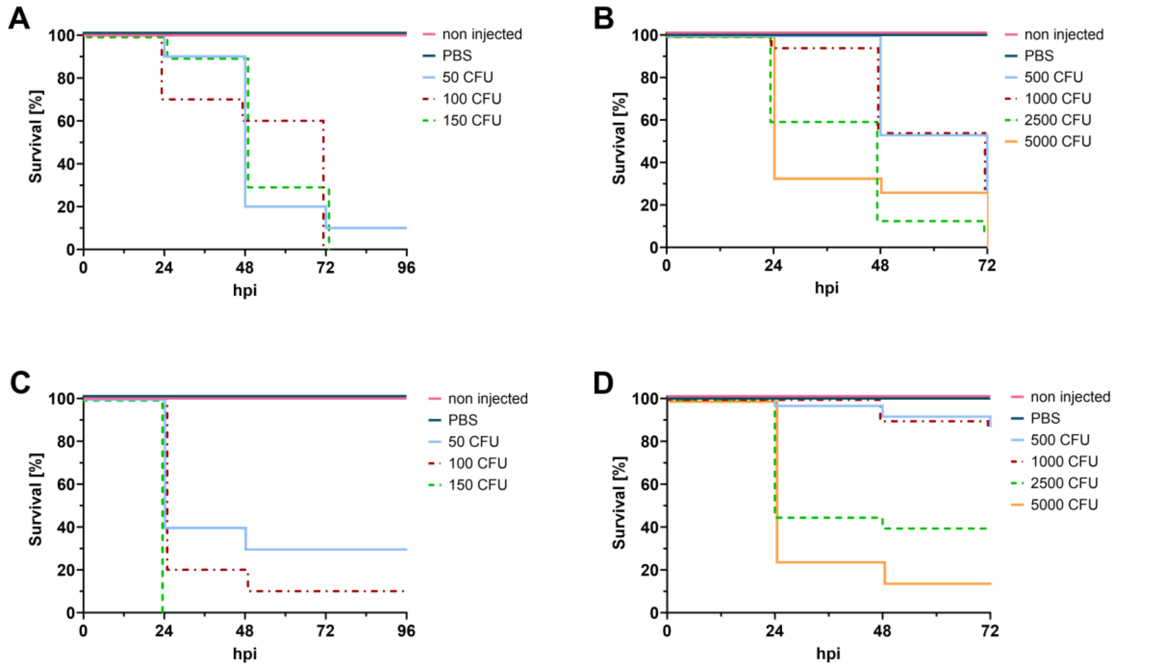

Figure S2. Zebrafish resistance to *Acinetobacter baumannii* infection is dependent on the developmental stage and the site of infection. Survival curves of zebrafish embryos infected with rising doses of *A. baumannii* ATCC17978 (n = 10–20) into the yolk sac at 1 dpf (A) and 2 dpf (B). Non-injected as well as PBS-injected embryos served as negative controls. Survival curves of zebrafish embryos infected with rising doses of *A. baumannii* into the caudal vein at 1 dpf (C) and 2 dpf (D). dpf: days post fertilization; hpi: hours post infection; CFU: colony-forming unit.

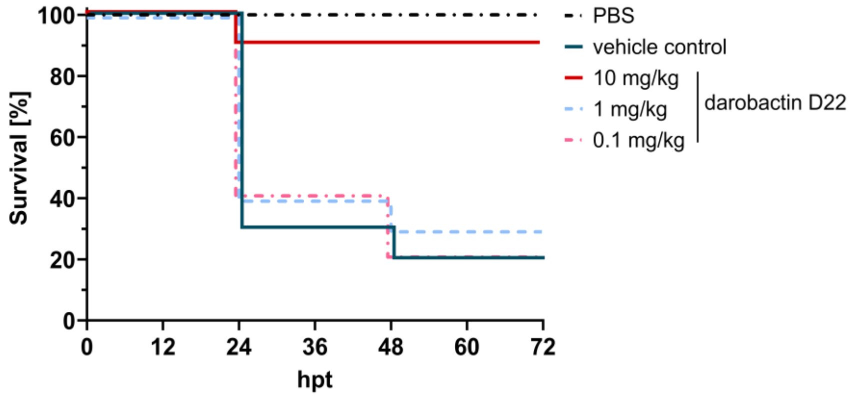

Figure S3. Dose titration of darobactin D22 in *Acinetobacter baumannii*-infected zebrafish embryos. Survival curves of zebrafish embryos following infection with *A. baumannii* at 2 dpf and treatment with rising doses of darobactin D22 (n = 10). Infected, PBS-treated embryos served as positive (vehicle) control, whereas non-infected PBS-injected embryos served as negative control. dpf: days post fertilization; hpt: hours post treatment.

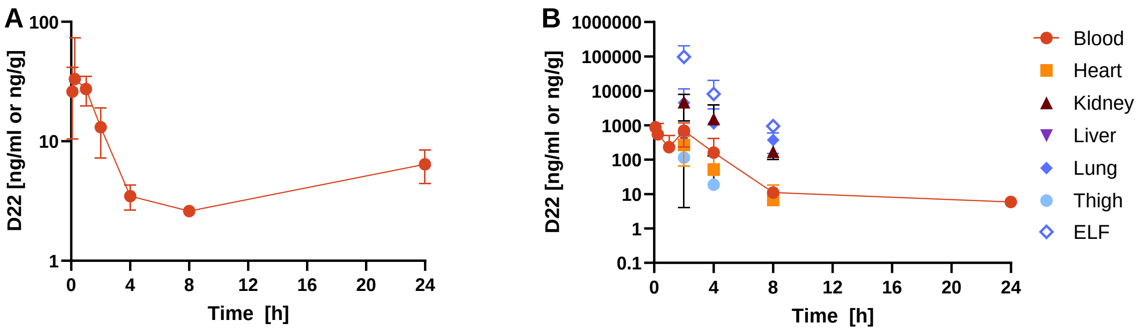

Figure S4. Pharmacokinetic studies in C57BL/6 mice. Blood levels of D22 after PO administration (20 mg/kg, A). Blood, ELF and tissue levels I heart, kidney liver, lung, thigh of D22 after intratracheal administration (5 mg/kg, B). Corresponding tissue levels are also given in Table S2. Concentrations are given in ng/mL for blood and ELF or in ng/g for heart, kidney, liver, lung and thigh tissue, representing means ± SD for 3 animals each. ELF: epithelial lining fluid.

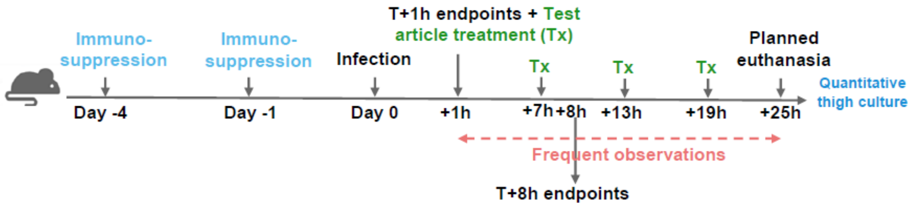

Figure S5. Experimental layout with treatment time points and duration for the neutropenic thigh infection model with *Pseudomonas aeruginosa* applying q6h dosing.

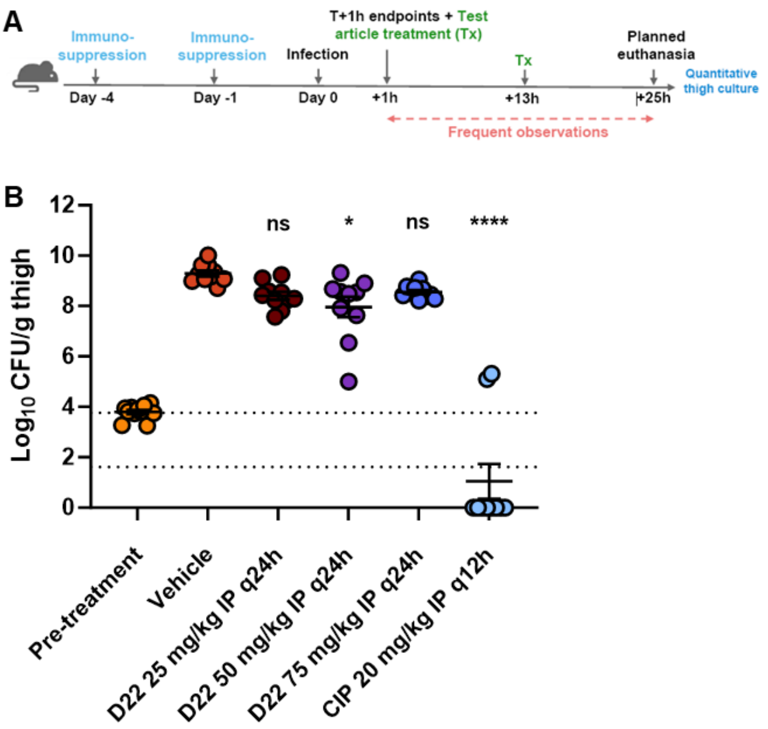

Figure S6. Murine neutropenic thigh infection model applying QID dosing with *Pseudomonas aeruginosa* PAO1. Experimental layout with treatment time points and duration (A) and bacterial burden in thigh muscle after 25 h of treatment (B). Dashed lines indicate the detection limit (1.4 log CFU/g) and stasis level (3.8 log CFU/g). 10 anmials were used per group. Mean ± SEM is depicted and significant differences vs. vehicle are indicated: ****: $p < 0.0001$, *: $p < 0.05$, ns: not significant (ANOVA with Dunnett's multiple comparison test). CFU: colony-forming unit; CIP: ciprofloxacin.

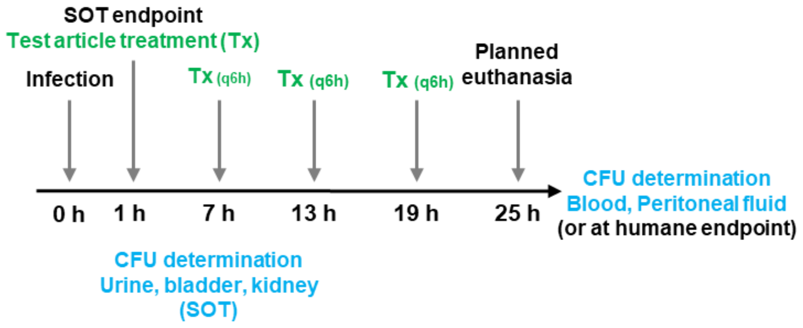

Figure S7. Experimental layout with treatment time points and duration for the peritonitis model with *Escherichia coli* 106-09 applying q6h and single dosing. SOT: start of treatment

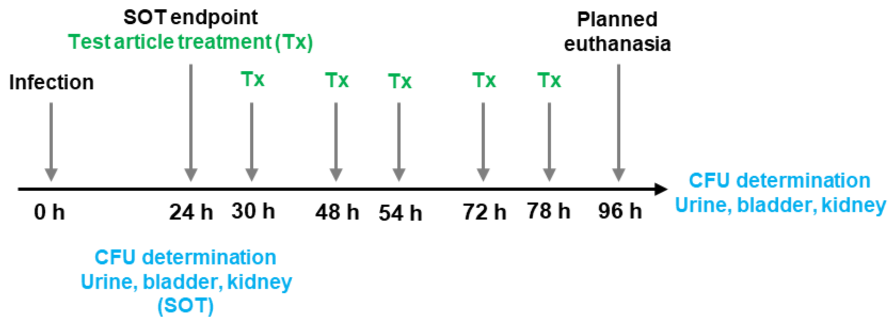

Figure S8. Experimental layout with treatment time points and duration for the urinary tract infection model with *Escherichia coli* C175–94 applying BID dosing. SOT: start of treatment

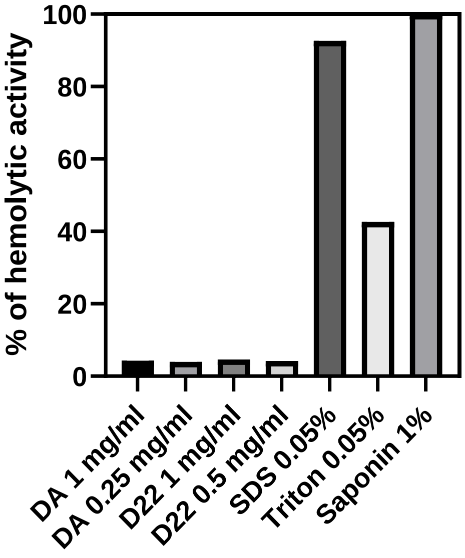

Figure S9. Hemolysis assay using human blood and darobactin A, D22 as well as positive controls SDS, triton and saponin. SDS: sodium dodecyl sulphate

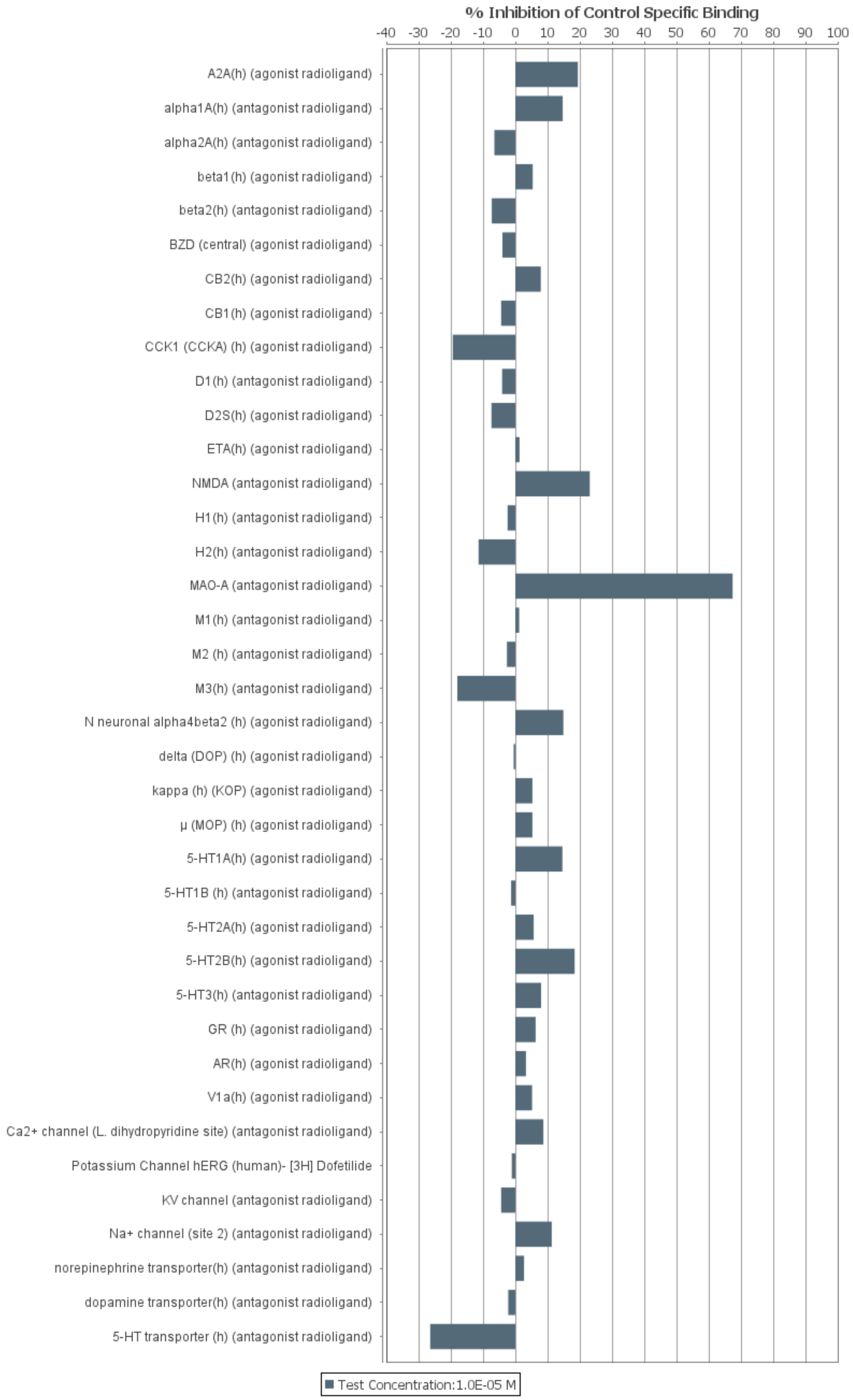

Figure S10. SafetyScreen44[TM] histogram for darobactin D22 at 10 µM final test concentration for binding assays representing means of 2 replicates.

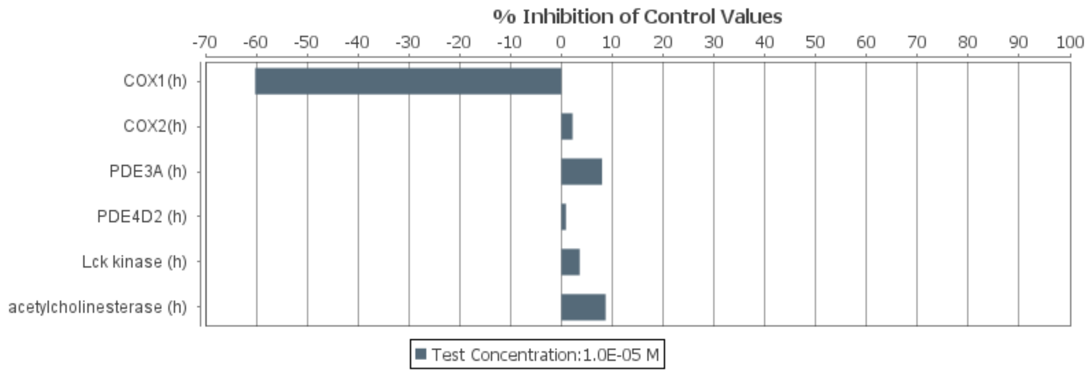

Figure S11. SafetyScreen44™ histogram for darobactin D22 at 10 µM final test concentration for enzyme and uptake assays representing means of 2 replicates.

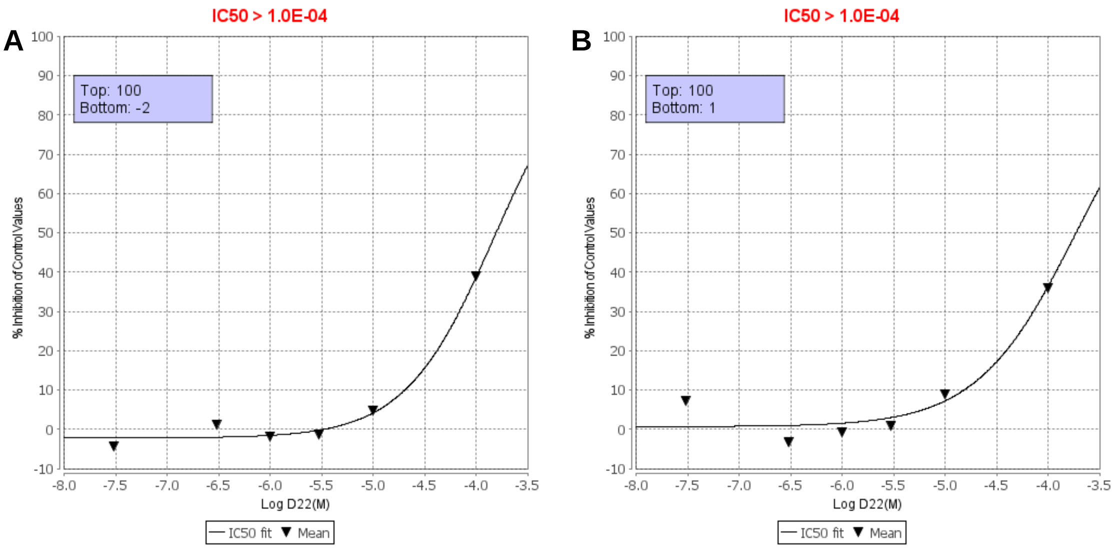

Figure S12. Functional inhibition assays of D22 against MAO-A (A) and MAO-B (B), resulting in 39 % and 36 % of inhibition at 100 µM, respectively. Values represent means of 2 replicates.

Supporting Tables

Table S1. MIC of D22, ciprofloxacin and colistin against isolates of *E. coli*. MIC determination was performed in technical duplicates. ND: not determined.

***E. coli* isolate**	**MIC [µg/mL]**			
	**Ciprofloxacin**	**Colistin**	**DA***	**D22**
ATCC25922	≤ 0.063	2–4	2	2
ATCC BAA-2340	> 32	2	2	2
ATCC BAA-2469	> 32	2	2	2
ATCC BAA-2471	> 32	1–2	2	1
CIP 107049	≤ 0.063	2–4	2	1
G260	> 32	2	2	1
G264	> 32	2	2	2
G38	8	2	2	1
G409	32	2	2	1–2
G41	> 32	2	2	1
G486	0.25	2	2	2
G487	0.063–0.125	1	2	2
NCTC13352	> 32	2–4	8	2
CIP 105706	> 32	1–2	2	2
G41	> 32	1	2	1–4
NCTC13846	> 32	8	2	0.5–4
PRAA1	32	1	2	1–4
PRAA17	> 32	2	2	1–4
PRAA18	> 32	2	4	4–8
PRAA21	> 32	2–4	2	2–4
R2670	> 32	1–2	2	1–4
R721	0.125	2	2	1–4
R995	> 32	2	2	1–4
R996	ND	1	2	1–8
CIP 104103	0.125	1–2	2	4–8
NCTC10418	0.125	2–4	1	0.25–4
NCTC13400	0.125	1	2	0.5–4
NCTC13441	> 32	2	2	0.5–1
PRAA3	UND	2–4	2	1–4
R1535	0.125	2	4	1–2

*DA reference data was generated in an independent experiment

Table S2. MIC of D22, ciprofloxacin and colistin against isolates of *P. aeruginosa*. MIC determination was performed in technical duplicates.

***P. aeruginosa* isolate**	**MIC [µg/mL]**			
	**Ciprofloxacin**	**Colistin**	**DA***	**D22**
PAO1	1	4	2	1
CIP 105250	0.125–0.25	2	16	8
NANC 01	32	2	32	8
NCTC13437	32	2	32	8

PBI 01	> 32	4	32	8
PBI 03G	32	2	64	16
PGP 02	> 32	4	32	16
CIP 105381	0.5	2	32	8
CIP 107051	0.125	2	32	8
ATCC BAA-2114	4	2	16	8
BOR 05	16	4	32	8
PBI 07	> 32	2	32	8
NANC 06	32	4	32	8
CLI 03	> 32	2	64	16
BOR 04	32	4	32	8
DIJ 02	32	2	16	4
CAE 02	4	2	32	8
CHRU LILLE VAP11	2	4	32	8
R1541	> 32	4	16	8
CHA	0.5	2	32	8
ROU 01	16	4	16	4
TOUL 01	32	2-4	32	8
CHRU LILLE VAP65a	0.25	4	32	16
CLI 02	2	2–4	64	8–16
SDE 05	0.5	2	32	8
PA 43	0.125	2	64	8
COL 03	> 32	2	32	8–16
STR 02	32	2	32	4–8
R2255	0.5	2	2	2
R2254	0.125	4	2	1
R2256	0.125–0.5	2	2	1

*DA reference data was generated in an independent experiment

Table S3. MIC of D22, ciprofloxacin and colistin against isolates of *A. baumannii*. MIC determination was performed in technical duplicates.

***A. baumannii* isolate**	**MIC [µg/mL]**			
	**Ciprofloxacin**	**Colistin**	**DA***	**D22**
ATCC17978	0.5	4	16	1
ATCC19606	1	4	32	4
ATCC27224	0.25	2	16	0.5
CIP 105742	0.125–0.25	2	8	0.5
CIP 107292	> 32	4–8	64	4–8
DSM 30008	0.25	4	16	1
G478 (AYE)	> 32	2–8	32	4
NCTC13301	> 32	8	64	4
R831	8	4–8	64	4–8
R835	32	4–8	64	4–8
ATCC19003	1-2	2-4	>64	8
ATCC19187	0.5	4	16	1–2
ATCC51432	16	8	>64	8

ATCC BAA-1605	> 32	2	32	2
ATCC BAA-1710	32	4–8	32	2
ATCC BAA-1791	> 32	2	16	0.5
ATCC BAA-1795	> 32	8	64	8
ATCC BAA-1878	4-8	4	64	4
ATCC BAA-747	0.5	4	8	0.5
BM4652 (efflux mutant)	1	4	4	0.5
BM4454	32	4–8	4	4–8

*DA reference data was generated in an independent experiment

Table S4. MIC of D22, ciprofloxacin and colistin against isolates of *K. pneumoniae*. ND: not determined. MIC determination was performed in technical duplicates.

***K. pneumoniae* isolate**	**MIC [µg/mL]**			
	**Ciprofloxacin**	**Colistin***	**DA***	**D22**
ATCC13883	< 0.063	1	2	2
ATCC13883 ColR_	< 0.063	64	4	8
ATCC43816	< 0.063	1	2	2–4
ATCC BAA-1705	> 32	2	4	4
ATCC BAA-1903	> 32	1	2	2–4
ATCC BAA-2342	32	4	4	8
ATCC BAA-2470	32	1	2	2
ATCC BAA-2472	> 32	2	4	4–8
ATCC BAA-2473	> 32	1	4	4–8
CIP 104119	ND	1	2	2
CIP 104298	0.125	16	2	2
CIP 105705	und	2	2	2
CIP 106982	1	2	4	4
G197	1	1	2	1–2
G201	< 0.063	1	4	2
G306	32	1	2	1
G52	32	1	2	2
Clinical Lyo-01	> 32	1	4	4
Clinical Lyo-02	1	1	4	4
Clinical Lyo-03	< 0.063	2	4	4
Clinical Lyo-04	0.25	1	2	2–4
Clinical Lyo-05	8	1	2	1
NCTC13438	> 32	1	4	4–8
NCTC13439	16	2	2	4
NCTC13442	4	1	2	2
NCTC13443	> 32	1	4	4
R1242	< 0.063	1	2	2
R1525	> 32	1	2	2
R2673	> 32	1	2	2
R750	ND	1	2	4

*DA and colistin reference data were generated in an independent experiment

Table S5. Minimum inhibitory concentrations of darobactin derivatives against WT and GFP-tagged *Acinetobacter baumannii* ATCC17978. MIC determinations were performed in duplicates.

	**MIC [µg/mL]**	
	**WT**	**GFP**
**Darobactin A**	16	16
**Darobactin D22**	4	4
**Darobactin D69**	2	1
**Ciprofloxacin**	1	1

Table S6. Tissue levels and tissue-to-blood ratios in the murine PK study after intravenous (IV), intraperitoneal (IP), subcutaneous (SC) and intratracheal (IT) administration. Concentrations are given in ng/mL (blood, ELF) or ng/g (heart, kidney, liver, lung, thigh); tissue-to-blood ratios in brackets (). Values represent means of 3 animals. BLQ = below limit of quantification.

**Tissue / Fluid**	**IV**		**IP**		**SC**				**IT**			
	**1 h**	**24 h**	**1 h**	**24 h**	**2 h**	**4 h**	**8 h**	**24 h**	**2 h**	**4 h**	**8 h**	**24 h**
Heart	615 (0.33)	BLQ	3995 (0.44)	28 (2.1)	724 (0.22)	147 (0.28)	44.5 (0.48)	15.7 (1.1)	267 (0.38)	51.2 (0.32)	6.72 (0.60)	BLQ
Kidney	12880 (7.0)	BLQ	45067 (5.0)	42 (45)	17920 (5.5)	5453 (10.3)	789 (8.5)	45.1 (3.3)	4672 (6.7)	1512 (9.4)	170 (15.3)	BLQ
Liver	465 (0.25)	BLQ	3052 (0.34)	BLQ	523 (0.16)	89.3 (0.17)	34.8 (0.37)	BLQ	397 (0.57)	46.8 (0.29)	BLQ	BLQ
Lung	2144 (1.2)	BLQ	12493 (1.4)	38 (41)	1457 (0.45)	231 (0.43)	64.7 (0.69)	9.23 (0.67)	4461 (6.4)	1121 (7.0)	372 (33)	BLQ
Thigh	309 (0.17)	54.0 (58)	1701 (0.19)	BLQ	307 (0.09)	47.2 (0.09)	37.9 (0.41)	BLQ	114 (0.16)	18.7 (0.12)	BLQ	BLQ
Blood	1843	0.927	9020	13	3263	531	93.2	13.8	694	161	11.1	6
ELF					3513 (1.1)	1255 (2.4)	204 (2.2)	NA	97092 (140)	8055 (50)	945 (85)	BLQ

Table S7. Urine concentrations and renal clearance calculation in the murine PK study based on 3 animals per group. Urine LLoQ 1 ng/mL. AUC: area under the curve; LLoQ: lower limit of quantification.

**Route**	**Time [h]**	**Urine conc. [ng/mL]**	**Sample volume [mL]**	**Conc. of parent in urine sample [ng]**	**Total parent in urine [ng]**	**Dose [ng]**	**% dose in urine**	**Blood AUC_{0-24} [ng*h/mL]**	**Weight [kg]**	**Renal clearance [mL/min/kg] (% of total clearance)**	**Blood clearance [mL/min/kg]**
IV	0-24	62000	2.94	182094	182094	333000	55	5946	0.022	7.7 (55)	14.0
IP	0-24	262000	1.14	297632	297632	1158000	26	38157	0.019		
SC	0-24	328000	3.23	1058784	1058784	1434000	74	29784	0.024		
IT	0-24	2030	0.74	1498	1498	288000	0.5	2243	0.019		

Table S8. Feces concentration and clearance calculation in the murine PK study based on 3 animals per group. Feces LLoQ 1 ng/mL. AUC: area under the curve; LLoQ: lower limit of quantification.

**Route**	**Time [h]**	**Feces conc. [ng/mL]**	**Sample volume [g]**	**Conc. of parent in feces sample [ng]**	**Total parent in feces [ng]**	**Dose [ng]**	**% dose in feces**	**Blood AUC_{0-24} [ng*h/mL]**	**Weight [kg]**	**Feces clearance [mL/min/kg] (% of total clearance)**	**Blood clearance [mL/min/kg]**
IV	0-24	625	0.48	298	298	333000	0.090	5946	0.022	0.04 (0.27)	14.0
IP	0-24	317	0.47	149	149	1158000	0.013	38157	0.019		
SC	0-24	822	2.45	2012	2012	1434000	0.140	29784	0.024		
IT	0-24	1830	1.27	2328	2328	288000	0.81	2243	0.019		

Table S9. MIC of D22 and gentamicin against clinical isolates of *Escherichia coli*. Compounds were tested in duplicate against each isolate.

**Isolate ID**	**MIC [µg/mL]**	
	**D22**	**Gentamicin**
E. coli DSA443	0.25–0.5	> 64
E. coli EC 106-09	0.5–1	1–2
E. coli C175-94	0.125–0.25	2
E. coli 2014-0162	1–2	64–> 64
E. coli 50639799	2	16–32
E. coli ATCC25922	1–2	0.5–1

Table S10. Difference in bacterial load in blood and PF compared to SOT in the *Escherichia coli* mouse peritonitis model. CC_{50}: concentration at which cell viability is reduced by 50%; PF: peritoneal fluid; SOT: start of treatment; MER: meropenem.

	**Δlog CFU/mL**	
	Blood	PF
D22 15 mg/kg IV q6h	–2.98	–4.05
D22 15 mg/kg SC q6h	–2.98	–4.33
D22 60 mg/kg SC	–1.96	–2.30
D22 2.5 mg/kg SC	0.03	–0.35
DA 2.5 mg/kg SC	3.38	2.07
Vehicle SC	4.79	4.35
MER 40 mg/kg SC q6h	–2.98	–4.33

Table S11. Darobactin D22 is non-toxic against a range of human cell lines. CC_{50}: concentration at which cell viability is reduced by 50%.

	**CHO-K1**	**HepG2**	**HCT-116**	**HeLa**	**Hep-2**	**U2 OS**	**KB3.1**	**A549**	**HEK293**
CC_{50} [µg/mL]	> 37	> 37	> 37	> 37	> 37	> 37	> 37	> 37	> 37

Materials and Methods

Production of darobactin A, D22 and D69. Darobactin A, D22 and D69 were produced and purified as described in Seyfert *et al.*[1] Moreover, D22 production was scaled up to technical scale fermentation (Frings PROREACT_17_P, Rheinbach, Germany) of 225 L (150 L working volume). For inoculation two preculture steps in shake flasks with LB-Medium (10 g/L casein peptone, 5 g/L yeast extract, 5 g/L sodium chloride, 50 mg/L kanamycin A, pH 7.0) are required. First preculture was inoculated with 0.02 % cryogenic culture. After 8 h at 37 °C and, 180 rpm, second preculture was inoculated at OD 0.1 for further 16 h incubation at 30 °C and, 180 rpm. The production (12.54 g/L dipotassium phosphate, 12 g/L yeast extract, 5 g/L sodium chloride, 4 g/L glucose monohydrate, 2.31 g/L monopotassium phosphate, 1 g/L ammonium chloride, 0.49 g/L magnesium sulphate hemihydrate, 50 mg/L kanamycin A 1 mg/L Vitamin B12, pH 7.6) was inoculated at OD 0.02. The fermenter is operated at 0.2 bar over pressure with the following parameters: pO2 controlled at of 0.2 and pH controlled at 7.6. When the culture reached OD 0.6 production was induced with 100 µM IPTG. After 72 h the culture was harvested, cells separated (CEPA centrifuge, Lahr/ Schwarzwald, Germany) and discarded. Darobactin 22 was secreted so that the culture supernatant was used for purification. First, product capture was performed in a fluidized bed for 1.5 h using 1 % absorbent resin XAD™ 16N. The resin was then transferred to a column and washed first with 10 bed volumes (BV) water and second with 10 BV of a mixture of acetone-deionized water (10 %/90 %), followed by 100 % methanol (5 BV), and a methanol-water mixture (80 %/20 %, 5 BV). Product elution occurred with 7 BV methanol-water (80 %/20 %)+0,1% formic acid (FA). The obtained freeze-dried crude product was subsequently submitted onto a C18 RP-MPLC Büchi Pure C-815 FlashPrep system (Büchi Labortechnik, Flawil Switzerland) with a Kronlab ODS-AQ C18 column (480 × 30 mm, 15 µm; YMC Europe GmbH, Dinslaken, Germany), UV: 220 nm, solvent A: water+0.2 % FA, solvent B: acetonitrile+0.2 % FA, 30 ml/min flow rate, gradient: 30 min 100 % solvent A, 90 min increasing to 12% solvent B, 60 min isocratic at 12 % solvent B. As a last step the darobactin 22 peak at 135 to 150 min was freeze-dried.

MIC_{50}/MIC_{90} determination. Briefly, MIC were performed in MHII at 37 °C. Dilution ranges of reference antibiotics were prepared 100X on DMSO or water and tested in a dose range with a two-fold dilution step. Dilution range was adapted according to reference antibiotics. 50 µl of MHII was distributed on 96 wells plate. 1 µl of each 100X antibiotics were distributed according to experiment plan. 50 µl of bacterial suspensions, prepared from fresh colonies on TSA, were added to obtain 5.10^5 CFU/ml final per well. Plates were incubated 20 to 24 hours at 37°C and OD_{600nm} measured with a plate reader (Spark reader Tecan). MICs, the minimal concentration of the compounds inhibiting growth, were calculated from Excel file data. Growth inhibition was defined as followed: ODmeasured ≤ ODmedium alone+10%. MICs determination by eyes was performed in parallel. Quality control was

validated with a purity check and an inoculum CFU determination. MIC_{90} determination was performed following CLSI guidance on panel of 20 to 30 strains. MIC_{50} and MIC_{90} represent the MIC value inhibiting the growth of respectively 50% and 90% of the strains tested.

MIC against Clinical Isolates of *E. coli*. The minimal inhibitory concentration (MIC) of D22 against clinical isolates of *E. coli* (Table S9) was determined at Statens Serum Institut in Copenhagen, Denmark following the CLSI guidelines.[2] Fresh (overnight) colonies, prepared from 5% horse blood agar plates, were suspended to 1.5×10^8 CFU/mL and further diluted in cation adjusted Mueller Hinton BBL II-broth (MH) broth to 1×10^6 CFU/mL and 50 µL was added to each well of the plate. Darobactin (D22) stock solution at 10 mg/mL was prepared by dissolving 2.2 mg of D22 in sterile water and 2-fold dilution was applied across the plate in duplicate against each isolate in the concentration range of 0.06 to 64 µg/mL.

Zebrafish Infection Model

<u>Bacterial strains and growth conditions:</u> *Acinetobacter baumannii* ATCC17978 was obtained from the American Type Culture Collection (ATCC) and grown in cation-adjusted Mueller Hinton broth (MHB2) at 37 °C. *A. baumannii* ATCC17978 expressing GFP (pWH1266-GFP) was obtained from Anton Y. Peleg, Monash University, Melbourne, and grown in MHB2 supplemented with 150 µg/mL carbenicillin (Carl Roth, Karlsruhe, Germany).[3]

<u>Antibiotic activity (minimum inhibitory concentrations):</u> Darobactin and ciprofloxacin (Sigma-Aldrich, St. Louis, MO, USA) stock solutions were prepared in MQ water. Minimum inhibitory concentrations (MICs) against *A. baumannii* (Table S5) were determined using the broth microdilution method according to EUCAST guidelines (ISO 20776-1:2019). In short, serial two-fold dilutions of antibiotics (0.03125 to 64 µg/mL) were prepared in 150 µL of MHB2 in sterile 96-well plates. Equal volume of the bacterial suspension was added and the plates were incubated at 37 °C for 18 h. The MIC was defined as the lowest concentration of the antibiotic with no visible growth of the microorganism.

<u>Zebrafish lines and maintenance:</u> Husbandry of adult zebrafish was performed according to internal guidelines set out in the German Animal Welfare Act (§11 Abs. 1 TierSchG). Experiments were carried out with wild type AB (obtained from the European Zebrafish Resource Center at Karlsruhe Institute of Technology) embryos within the first 120 hours post fertilization (hpf) as these early life stages are not considered as animal experiments according to the EU Directive 2010/63/EU.[4] Embryos were maintained in fresh 0.3x Danieau's (17.4 mM NaCl, 0.21 mM KCl, 0.12 mM $MgSO_4$, 0.18 mM $Ca(NO_3)_2$, 1.5 mM HEPES, 1.2 µM methylene blue, pH 7.1-7.3) at 28 °C. At a maximum of 120 hpf embryos were euthanized by submersion in ice water.

Microinjection of zebrafish embryos: Cultures of *A. baumannii* ATCC17978 GFP were grown until logarithmic phase (OD_{600} 0.4-0.8) and cells were harvested by centrifugation at 4,000 rpm, 22 °C for 10 min. The pellet was washed twice with PBS and resuspended in 4 % polyvinylpyrrolidone 40 (Sigma-Aldrich) in PBS to achieve 2x the desired cell count. Subsequently, the suspension was diluted 1:1 with phenol red solution (Sigma-Aldrich). Heat-killed bacteria were obtained by incubating bacteria at 90 °C for 15 min. Pulled glass capillaries for microinjection were prepared using a micropipette puller (P-1000, Sutter Instrument, Novato, CA, USA).

Infection of zebrafish embryos was performed at 24 hpf or 48 hpf. Embryos were dechorionated using 1 mg/mL pronase solution (Roche, Basel, Switzerland) and anaesthetized through immersion in 945 µM tricaine (Sigma-Aldrich). For microinjection, a pulled glass capillary was filled with the bacterial suspension and fixed to the micromanipulator (M-152, Narishige, London, England). The tip was manually cut with a tweezer and the injection volume was calibrated to 4 nL by injecting single droplets into mineral oil on a microscale slide (Bresser, Rhede, Germany). Zebrafish embryos were infected by microinjecting 4 nL into the caudal vein or the yolk sac (n = 10-20 embryos per infectious dose). Inocula were confirmed by colony counts of infected embryos. Negative controls included non-injected embryos, PBS-injected embryos ("mock infection") and heat-killed bacteria.

Treatment of infected embryos (infected with 2.500 CFU into the caudal vein at 48 hpf) was performed 3 hours post infection (hpi) via microinjection into the caudal vein. Antibiotics were dissolved in PBS and methylene blue (Sigma-Aldrich) was used as tracer dye. The treatment dose (for comparative treatment) was set to 10 mg/kg (embryo weighing approximately 1.5 mg). Vehicle-injected embryos served as control ("mock treatment"). 15 embryos were used per group. Embryos were maintained in 0.3x Danieau's at 28 °C and monitored daily for survival until 72 hpi. Treatment experiments were performed in 3 independent biological replicates.

To determine the bacterial burden of dead zebrafish embryos, embryos were washed three times with sterile PBS followed by mechanical homogenization using a micropestle (Carl Roth). The homogenates were diluted and appropriate dilutions were plated on CASO agar. CFU counts were determined after 24 h of incubation at 37 °C.

Statistical analysis was performed using GraphPad Prism (Boston, MA, USA) version 10.0.2. Survival of zebrafish embryos was evaluated using the Kaplan-Meier method. Comparison between survival curves were made using the log rank (Mantel-Cox) test. Statistical significance was assumed at p-values below 0.05 ($p < 0.05$: *, $p < 0.01$: **, $p < 0.001$: ***, $p < 0.0001$: ****).

***In vivo* PK study.** The in-life phase and PK was done at Selvita, Zagreb, Croatia. All PK experiments were carried out in an Association for Assessment and Accreditation of Laboratory Animal Care International-accredited facility under the supervision of Selvita's institutional ethics committee (CARE-

Zg, Committee on Animal Research Ethics-Zagreb) in collaboration with the Croatian Competent Authority and the National Ethics Committee, granting this project a 5-year authorisation, in compliance with the 2010/63/EU Directive requirements.

Animals: The study was performed in male, 6–7 weeks old C57BL/6 mice. Animals were housed in a temperature-controlled room (22±2°C), relative humidity 55±10%, 15–20 air changes per hour, atrificial light cycle of 12 h light and had free access to potable water and food (SDS VRF 1 (P), UK).

Sampling: Serial or terminal blood samples were collected into EDTA-coated tubes at the following time points after administration: 0.05, 0.25, 0.5, 1, 2, 4, 8, 24 h (IV); 0.08, 0.25, 0.5, 1, 2, 4, 8 h (IP, PO); 0.25, 0.5, 1, 2, 4, 8, 24 h (SC); 0.08, 0.25, 1, 2, 4, 8 h (IT). Terminal tissue samples at 24 h were taken into Precellys® tubes for all animals. Additional, terminal tissue samples were taken at 1 h for the IV and IP groups. For the SC and IT groups, terminal tissue samples were further taken at 2, 4, 8 h. Urine and faeces were collected following IV, IP, SC and IT administration during the 0–24 h interval. Pooled samples (3 animals) were collected into emtpy tubes and kept chilled, followed by centrifugation at 3000 rpm, 10 min, 4°C. Total urine volume was recorded. For the SC and IT routes, bronchoalveolar lavage (BAL) fluid was collected from 3 animals at 2, 4, 8 and 24 h (terminal sampling) via cannulating the trachea using a sterile, luer fitting 1.0 x 130 mm cannula. Lungs were lavaged with 3 columes of PBS (1 mL in total). Collected batches were centrifuged (5 min, 3500 rpm, 4°C).

Bioanalysis: Blood, urine, faeces, BAL and tissue concentrations were determined using a LC-MS/MS. Protein precipitation after tissue homogenization for tissue samples with organic solvent was done before sample preparation. Blank mouse plasma was used for preparation of blank samples, calibrations and quality control samples. At least six calibration standards were prepared. The concentration in ELF was determined according to Rodvold *et al*.[5] Pharmacokinetic analysis was performed using WinNonlin Phoenix® software (Certara, version 8.3) from the mean animal concentration per time-point, non-compartmental analysis and the target dose.

***In vivo* thigh infection model.** The murine neutropenic thigh infection model was performed at the facilities of Evotec (UK) Ltd, UK. All animal experiments were performed under UK Home Office Licences and with local ethical committee clearance. The Evotec (UK) animal facility holds a UK Home Office Establishment Licence, is fully AAALAC-I accredited and is a dedicated Containment Level 2 facility. All experiments were performed by trained scientists that had completed parts A, B and C of the Home Office Personal Licence course and held a current personal licence.

Specific pathogen-free mice (approximately 11 to 15 g) supplied by Charles River (Margate, UK) were housed in autoclaved individual ventilated cages that provided the mice with HEPA-filtered air. Mice had free access to food, water, aspen chip bedding, nesting material, and shelters. Mice were acclimatised to the animal facility before the start of the study. Appropriate measures were taken to

reduce stress and harm arising from experimental procedures, including use of analgesia, access to wet food and additional bedding. The room temperature, relative humidity and maximum background noise were maintained according to Home Office Code of Practice. Mice were exposed to 12-hour light/dark cycles.

Mice were immunosuppressed using cyclophosphamide administered subcutaneously (SC) at 150 mg/kg on day minus 4 and at 100 mg/kg on day minus 1 relative to the day of infection. Following induction of anaesthesia with isoflurane, mice (approximately 30 g) were infected with 1.8×10^3 CFU/thigh *P. aeruginosa* PAO1 by intramuscular (IM) injection of 50 µL bacterial suspension into both thighs. Following infection, a single dose of carprofen was administered SC to provide pain relief.

One hour post-inoculation, one group of mice were euthanized to quantify the pre-treatment bacterial burden. Q6h treatment was then initiated and mice were injected with either 25, 30, or 50 mg/kg/dose of Darobactin formulated in saline for injection. Mice were observed frequently throughout the study. At eight or 25 hours post infection, mice were euthanized by overdose of pentobarbitone and death confirmed by cervical dislocation. Thighs were dissected and individually homogenized in ice-cold sterile phosphate-buffered saline, before being serially diluted in PBS and plated onto Pseudomonas CN selective agar (Oxoid, UK). The bacterial burden was quantified following overnight incubation of agar plates at 37°C. For analytical purposes, burdens from individual thighs were counted as independent data points.

***In vivo* peritonitis model.** The murine peritonitis/sepsis model was conducted at Statens Serum Institut in Copenhagen, Denmark, under the supervision of the Danish Animal Ethical Council. Ethics approval was obtained (license 2019-15-0201-00019). The study was performed as described previously.[6] In brief, six to seven weeks old CD-1 female mice were acquired from Envigo (Netherlands) and kept in cages of eight and acclimatized for a week before the study and allowed free access to chow and water. Mice were inoculated intraperitoneally with 10^5 CFU in 0.5 mL of sterile saline with 5 % porcine mucin.

One hour post inoculation, q6h treatment was initiated and mice were injected with 0.2 ml of Darobactin (D22) formulated in pharmaceutical grade solution of physiologic saline and injected subcutaneously or intravenously. Mice were observed during the study and scored 0 – 4 based on their behavior and clinical signs of discomfort and euthanized at the humane endpoint as a surrogate marker for death.

After mice were sacrificed, blood was obtained by cardiac puncture and peritoneal fluid lavage was recovered. Both blood and peritoneal fluid were immediately processed for quantitative counts by applying 20 µL spots of ten-fold serial dilutions on 5% horse blood agar plates.

***In vivo* urinary tract infection model.** The murine urinary tract infection model was conducted at Statens Serum Institut in Copenhagen, Denmark under the supervision of the Danish Animal Ethical Council. Ethics approval was obtained (license 2020–15-0201–00730).

Bacterial challenge organism: The challenge organism was *Escherichia coli* C175-94 (serotype O8:K48:H4), a clinical isolate elaborating type 1 fimbriae, previously used for developing and validating the urinary tract infection model.[7]

Animals: Upon arrival to Statens Serum Institut animal facility, female C3H/HeNHsd mice 5-6 weeks old (Envigo, Netherlands), were randomized to 8 mice per cage and acclimatized 1 week before experimental start. Bedding was from Tapvei and Enviro-Dri nesting material and cardboard houses (Bio-serv) was offered as enrichment. Mice had free access to domestic quality drinking water and food (Teklad Global diet 2916C-Envigo) and peanuts and sunflower seeds (Køge Korn A/S). All animal experiments were approved by the National Committee of Animal Ethics, Denmark, and adhered to the standards of EU Directive 2010/63/EU. Mice were monitored twice daily for clinical symptoms of infection or discomfort and euthanized if reaching humane endpoints specified in the ethical permission (2020-15-0201-00730).

Inoculation, treatment and sampling: This model has been described and validated previously.[7,8] Three days before start of study and during the study the mice have free access to 5 % glucose as drinking water. In brief, mice bladders were emptied by gently pressing their abdomen. Mice were then anaesthetized by subcutaneous administration of a combination of Zoletil and Torbugesic (0,1 mg/ml). Overnight *E.coli* colonies were suspended in saline to 109 cfu/mL and bacterial 0.05 mL suspension was inoculated slowly transurethrally by use of plastic catheters (Polyethylene catheter, Becton Dickinson, cat. 427400, 0.28x0.61 mm) into the bladder. On day 1, 2 and 3 post inoculation, mice were treated subcutaneously with 0.2 mL of test compound at 9 am and 3 pm. On day 1 and day 4 post inoculation, urine was sampled and mice were sacrificed by cervical dislocation and bladder and kidneys were aseptically removed. The bladder and kidneys were stored at -80° C and later homogenized with steel beads on a tissuelyser in 0.5 mL or 1 mL saline respectively. Colony counts of urine were determined immediately. All samples were 10-fold diluted in saline and 20-µL spots were applied on agar plates in duplicates. All agar plates were incubated 18-22 h at 35°C in ambient air.

Hemolysis study. Whole human blood from anonymous donors provided by the French blood establishment (EFS) was shaken at 20 rpm, 37°C for 10 min before the assay. 200 µL of blood, 200 µL of PBs and 8 µL of test or reference compounds at 50X the final concentration were mixed and incubated 45 min at 37°C and then centrifudes for 5 min (700 g). OD_{540nm} of the supernatant was measured and the hemolytic % was calculated following the formula: (OD test compound – OD blank)-(OD positive

control-OD blank)x100. Saponin was used as a positive control (100% lysis), SDS and Triton were used as positive controls for partial hemolysis and PBS as a negative control.

Off-Target Safety Screen44^{TM} Panel. The safety screen panel and functional follow-up assays for inhibition of MAO-A and MAO-B were performed by the CRO Eurofins Cerep according to their guidelines. In each experiment and if applicable, the respective reference compound was tested concurrently with the test compounds, and the data were compared with historical values determined by Eurofins. The experiment was accepted in accordance with their Standard Operating Procedures.

Cytotoxicity. All tested cell lines were obtained from the German Collection of Microorganisms and Cell Cultures (DSMZ) or the American Type Culture Collection (ATCC) and were maintained at 37 °C with 5 % CO_2 in growth medium containing 10 % FBS as specified by the depositor. For cytotoxicity testing,120 µL cell suspension per well (5 x 10^4 cells/mL) was seeded into 96-well plates (CellBind® surface) and incubated for 2 h. Serial dilutions of test compounds (0.63 ng/mL to 37 µg/mL) were prepared in in culture medium and added (60 µL) to the cells. Doxorubicin served as positive control. Cells were treated for 120 h. To assess viability, 20 µL MTT reagent (5 mg/mL in PBS) was added to each well and incubated for 2 h. The wells were emptied and 100 µL isopropanol/10 N HCl (1000:4) per well was added. Plates were analyzed by measuring the absorbance at 570 nm (plate reader Infinite® 200 Pro, Tecan). After normalization of data to the respective solvent control, the calculated percentage of growth inhibition was plotted using GraphPad Prism software (version 10.0.2).

References

1 Seyfert, C. E.; Müller, A. V.; Walsh, D. J.; Birkelbach, J.; Kany, A. M.; Porten, C.; Yuan, B.; Krug, D.; Herrmann, J.; Marlovits, T. C.; Hirsch, A. K. H.; Müller, R. New Genetically Engineered Derivatives of Antibacterial Darobactins Underpin Their Potential for Antibiotic Development. *J. Med. Chem.* **2023,** *66*, 16330–16341. DOI: 10.1021/acs.jmedchem.3c01660.

2 Clinical and Laboratory Standard Institute (CLSI). Performance Standards for Antimicrobial Susceptibility Testing 2018. www.clsi.org.

3 Bhuiyan, M. S.; Ellett, F.; Murray, G. L.; Kostoulias, X.; Cerqueira, G. M.; Schulze, K. E.; Mahamad Maifiah, M. H.; Li, J.; Creek, D. J.; Lieschke, G. J.; Peleg, A. Y. Acinetobacter baumannii phenylacetic acid metabolism influences infection outcome through a direct effect on neutrophil chemotaxis. *Proc. Natl. Acad. Sci. U. S. A.* **2016,** *113*, 9599–9604. DOI: 10.1073/pnas.1523116113.

4 Strähle, U.; Scholz, S.; Geisler, R.; Greiner, P.; Hollert, H.; Rastegar, S.; Schumacher, A.; Selderslaghs, I.; Weiss, C.; Witters, H.; Braunbeck, T. Zebrafish embryos as an alternative to animal experiments--a commentary on the definition of the onset of protected life stages in animal welfare regulations. *Reprod. Toxicol.* **2012,** *33*, 128–132. DOI: 10.1016/j.reprotox.2011.06.121.

5 Rodvold, K. A.; George, J. M.; Yoo, L. Penetration of anti-infective agents into pulmonary epithelial lining fluid: focus on antibacterial agents. *Clin. Pharmacokinet.* **2011,** *50*, 637–664. DOI: 10.2165/11594090-000000000-00000.

6 Vingsbo Lundberg, C.; Vaara, T.; Frimodt-Møller, N.; Vaara, M. Novel polymyxin derivatives are effective in treating experimental Escherichia coli peritoneal infection in mice. *J. Antimicrob. Chemother.* **2010,** *65*, 981–985. DOI: 10.1093/jac/dkq072.

7 Hvidberg, H.; Struve, C.; Krogfelt, K. A.; Christensen, N.; Rasmussen, S. N.; Frimodt-Møller, N. Development of a long-term ascending urinary tract infection mouse model for antibiotic treatment studies. *Antimicrob. Agents Chemother.* **2000,** *44*, 156–163. DOI: 10.1128/AAC.44.1.156-163.2000.

8 Jakobsen, L.; Lundberg, C. V.; Frimodt-Møller, N. Ciprofloxacin Pharmacokinetics/Pharmacodynamics against Susceptible and Low-Level Resistant Escherichia coli Isolates in an Experimental Ascending Urinary Tract Infection Model in Mice. *Antimicrob. Agents Chemother.* **2020,** *65. DOI:* 10.1128/AAC.01804-20.
